# COVID‐19 Infection Is Associated With Higher Stress in Urban American Indian and Alaska Native Peoples: Results From a Cross‐Sectional Survey

**DOI:** 10.1002/brb3.71783

**Published:** 2026-09-17

**Authors:** Austin Henderson, Richard MacLehose, Spero M. Manson, Dedra Buchwald

**Affiliations:** ^1^ Department of Economics University of Otago Dunedin New Zealand; ^2^ Institute For Research and Education to Advance Community Health Elson S Floyd College of Medicine Washington State University Pullman Washington USA; ^3^ Department of Epidemiology and Community Health University of Minnesota School of Public Health Minneapolis Minnesota USA; ^4^ Centers For American Indian and Alaska Native Health University of Colorado Anschutz Medical Campus Aurora Colorado USA; ^5^ Department of Neurological Surgery University of Washington Medical School Seattle Washington USA

**Keywords:** Alaska Native, American Indian, COVID‐19, indigenous, stress

## Abstract

**Introduction:**

American Indian and Alaska Native (AI/AN) communities experienced a disproportionate burden of COVID‐19, including among the highest rates of infection, hospitalization, and mortality in the United States. The pandemic also introduced widespread stressors, and elevated perceived stress is itself a risk factor for adverse physical and mental health outcomes. Little is known about whether COVID‐19 infection is associated with perceived stress among AI/AN peoples. We examined the association between self‐reported COVID‐19 infection and perceived stress among urban AI/AN adults.

**Methods:**

Self‐report surveys were initiated by *N* = 788 patients of 5 geographically dispersed health organizations that primarily served AI/AN peoples in urban settings between January and May of 2021. COVID‐19 infection was assessed by participants’ self‐report of either a positive test result or having been informed they were infected by a health professional. Stress was measured using the Perceived Stress Scale‐4 (PSS‐4). The relationships between COVID‐19 infection and mean stress were assessed using linear regressions; the relationships between COVID‐19 infection and specified stress levels were assessed using modified Poisson regressions with results expressed as relative risk (RR).

**Results:**

COVID‐19 infection was associated with an adjusted *β* = 0.9 (95% CI: 0.1, 1.8) points higher mean PSS‐4 score and RR = 3.2 (95% CI: 1.2, 8.5) of high (≥13) PSS‐4 score.

**Conclusions:**

COVID‐19 infection was associated with elevated perceived stress among urban AI/AN adults; this association was robust to adjustment for comorbid health conditions, social determinants of health, healthcare access, and pandemic‐related stressors. Healthcare providers should be aware that AI/AN people who have been infected with COVID‐19 are at higher risk of elevated stress, and further research on the downstream health consequences of stress among those infected with COVID‐19 is warranted.

## Graphical abstract

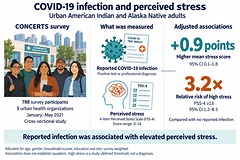

## 1 Introduction

Stress is a complex response to a challenging condition or change (Hammen et al. 2009) that involves interrelated biological and psychological mechanisms and can contribute to adverse health consequences. Stress can directly affect a wide array of health conditions (Steptoe and Kivimäki 2012, Moreno‐Smith et al. 2010) and, in particular, high perceived stress levels before contracting COVID‐19 have been associated with greater risk of developing long‐term COVID‐19 health conditions, like shortness of breath or persistent cough, that last for more than 4 weeks (Wang et al. 2022). There is, as of yet, a limited understanding of how contracting COVID‐19 could lead to elevated stress, but there are several plausible and nonexclusive mechanisms. For example, while the etiology of COVID‐19‐related psychoneurological damage is unclear, the physical damage to the brain and nervous system caused by the disease could lead to persistent feelings of anxiety, a construct closely related to stress, and a frequently studied component of long‐COVID (Nalbandian et al. 2021, Thye et al. 2022). COVID‐19 infection has been shown to be positively associated with negative mental health in diverse populations (Badinlou et al. 2024, Rivas Nieto et al. 2026), including Indigenous people in Brazil (Pereira et al. 2026), though little is known about the association between COVID‐19 infection and stress in American Indian and Alaska Native (AI/AN) peoples. In this study, we examine the associations between stress and COVID‐19 infection in AI/AN individuals.

In addition to negative effects in individuals, stress may contribute to health inequalities, as previous studies have shown that stress is unequally distributed by social class (Kahneman and Deaton 2010). The COVID‐19 pandemic in general has exacerbated pre‐existing inequalities (Van Dorn et al. 2020, Perry et al. 2021) and introduced numerous additional stressors into people's lives (Park et al. 2020). In particular, the COVID‐19 pandemic has had severe consequences for AI/AN communities, who experienced widespread disease incidence, hospitalizations, and mortality (Burki 2021, Manson and Buchwald 2021, Goldman and Andrasfay 2022, Leggat‐Barr et al. 2021). It should be noted that AI/AN peoples demonstrated exceptional uptake of COVID‐19 preventive measures. including testing, vaccination (Silberner 2021), and Tribal protocols to prevent spread, including restricting access to Tribal land. These individual‐ and community‐level responses highlight the speed at which AI/AN peoples were willing and able to respond to a devastating pandemic. Evidence suggests that the high burden of COVID‐19 in AI/AN communities was driven by higher prevalence of comorbidities (Goldman and Andrasfay 2022) and adverse social determinants of health (Leggat‐Barr et al. 2021). Given the impact of COVID‐19 in these communities, further study into the effects of COVID‐19 in these populations is especially warranted.

To study the associations between stress and COVID‐19 infection in AI/AN peoples, we surveyed AI/AN patients at 5 health organizations that primarily serve AI/AN peoples in urban settings in 5 states across the USA between January and May of 2021. This study helps overcome the challenge of the absence of relevant data on AI/AN peoples, which has been noted by the Centers for Disease Control and Prevention as an important public health gap (Hatcher et al. 2020). This absence of data is particularly severe for urban AI/AN peoples, who comprise approximately 75% of the total AI/AN population (U.S. Census Bureau 2012). While not representative of all AI/AN peoples and experiences, this survey constitutes the most comprehensive and geographically inclusive data to date with respect to stress in these populations.

## 2 Methods

### 2.1 Study Design

Community Organizations for Natives: COVID‐19 Epidemiology, Research, Testing, and Services (CONCERTS) was designed to increase COVID‐19 testing and elucidate health disparities related to COVID‐19 among urban AI/AN peoples. We created and implemented a cross‐sectional patient survey in partnership with 5 healthcare organizations that primarily serve AI/AN peoples in urban settings to identify barriers, facilitators, attitudes, and risk factors for COVID‐19 testing and vaccination. These health organizations provide a range of services including outreach and referral, dental, primary, and behavioral health care, as well as health promotion and disease prevention supported by state, federal, and local resources. They serve Albuquerque, NM, Anchorage, AK, Denver, CO, Minneapolis‐St. Paul, MN, and Wichita, KS. Their active patient populations in 2019 ranged from 1,269 to 25,043 unique patients. This study was approved by the Washington State University Institutional Review Board (#18590), the Alaska Area Institutional Review Board (#2020‐11‐044), and Tribal research review committees at Southcentral Foundation (SCF) and the Alaska Native Tribal Health Consortium. Tribal approval was also obtained before dissemination.

### 2.2 Eligibility and Recruitment

Patients eligible for inclusion in CONCERTS were seen at any of the 5 health organizations within the year before the survey, were 18 years or older, AI/AN, and not diagnosed with dementia or other serious cognitive issues (ICD‐10 codes F01‐04, G30, or G31). To ensure we enrolled enough older participants at highest risk of severe COVID‐19, we used stratified sampling by age (18–54 years vs. 55 years and older). Each clinic generated a list of eligible participants from its electronic health records, randomly sampled 200 in each age group, and invited them to participate using a multipronged strategy. Clinic patients with an email address in their respective health organization's electronic health records were sent an invitation to participate in CONCERTS with a link to an online REDCap survey; those without an email address were mailed invitations. Patients received up to 4 email or phone call reminders depending on the data collection mode over 14 days. Our goal was to enroll at least 150 participants per clinic. If this sample size was not achieved through the first round of emails, letters, and phone calls, we selected a new random sample of eligible patients from the electronic health record of the clinic in question. Participants were enrolled during the COVID‐19 pandemic from January to May 2021 and were compensated $100. Of 4603 eligible patients contacted, 788 (17.1%) initiated the survey and thus comprised the study population. All participants gave informed consent before beginning the questionnaire.

### 2.3 Survey Development and Measures

The survey collected information about patients’ demographic characteristics, comorbid health conditions, individual and structural social determinants of health, access to health services, pandemic impact on physical and mental health, socioeconomic and quality of life status, experience with and attitudes toward COVID‐19, and receipt of and structural barriers to COVID‐19 testing. Survey questions were developed based on the NIH RADX‐UP Common Data Elements (Tromberg et al. 2020) and PhenX Toolkit (Hamilton et al. 2011) when possible and appropriate. After the survey was developed, experts at each healthcare organization provided feedback on the appropriateness and relevance of all survey questions to their clinic populations. The resulting survey had 164 questions, which took about 40 min to complete.

### 2.4 Measures

Perceived stress was measured using the 4‐item perceived stress scale (PSS‐4) (Cohen 1988), a shortened version of the original scale validated by Cohen et al. (1983). The PSS‐4 is a common 4‐item self‐report instrument that uses a 5‐point response scale for each item (0 = never, 1 = almost never, 2 = sometimes, 3 = fairly often, 4 = very often). Individual items were summed to create a score from 0 to 16. Items were designed to detect how unpredictable, uncontrollable, and overwhelming respondents felt their lives had been in the last month. The PSS‐4 is reliable when administered to diverse populations (Cohen et al. 1983). Our sample showed good internal reliability, with Cronbach's alpha = 0.70. The summed PSS‐4 score is hereafter referred to as perceived stress.

COVID‐19 infection was a constructed binary variable defined as positive when a participant responded affirmatively to either of two questions. The first question was whether the participant had ever been told by a doctor, nurse, or other healthcare professional that s/he was infected with or had COVID‐19; the second was whether s/he had ever tested positive for COVID‐19.

If participants responded affirmatively to having tested positive for COVID‐19, they were asked what the date of their first positive test was. A variable, days since first positive test, was then calculated as the difference between that date and the date the participant completed the survey.

Definitions for all covariates are presented in the Appendices, and all survey questions are presented in their entirety in the Supporting Online Materials.

### 2.5 Primary Analytic Approach

Because the data are cross‐sectional, these models are intended to identify associations between reported infection and current stress levels, rather than to establish a temporal or causal sequence. To examine the association between COVID‐19 infection and mean stress, we first fit 2 linear regressions with perceived stress as the dependent variable and COVID‐19 infection as the independent variable. The first regression, model (1), only includes COVID‐19 infection as an explanatory variable, and the second regression, model (2), adjusted for gender, household income, education, and age. These covariates were chosen due to their plausible connection with both COVID‐19 infection and stress, and we also conducted sensitivity analyses with other covariates (see Section 2.6). In both regressions, we also included site fixed effects for each health organization, with the coefficients not reported here to avoid direct comparisons between sites. Regression coefficients β are presented for COVID‐19 infection, along with 95% confidence intervals, and can be interpreted as the adjusted mean increase in perceived stress levels associated with a change in the specified variable.

We also estimated the association between days since first positive COVID‐19 test and stress among participants who had a positive COVID‐19 test using a linear regression, also adjusting for gender, household income, education, and age. To further assess whether the time elapsed between infection and survey completion influenced perceived stress, we classified COVID‐19‐positive participants by the clinical phase of illness corresponding to the time since their first positive test, acute (≤4 weeks), ongoing symptomatic (4–12 weeks), and post‐COVID‐19 (>12 weeks) (Soriano et al. 2022), and compared adjusted mean perceived stress across these categories using linear regression, also adjusting for gender, household income, education, age, and site.

Second, we conducted an analysis of the association between COVID‐19 infection and two thresholds of perceived stress. For both thresholds, we did this using a multivariable modified Poisson regression (Zou 2004), with an outcome equal to 1 if the threshold was met, and 0 if the threshold was not. Estimates are presented as relative risk (RR) with 95% confidence intervals. As in the linear regressions above, in model (1), we only include COVID‐19 infection as an explanatory variable, and in model (2), adjusted for gender, household income, educational attainment, and age. We first tested a lower threshold of ≥6, as 6 was the observed overall mean perceived stress score. We then tested a higher threshold of ≥13, as this corresponds to the highest quartile of possible scores, an approach comparable to that of Wang et al. (2022), who also used quartiles (but with a threshold of 7 corresponding to the highest quartile in their sample) in their study of perceived stress and risk of post‐COVID‐19 conditions.

All analyses were conducted using inverse probability weights. Weights were generated to correct for sample selection based on age and nonresponse according to age and gender compared with the overall patient population at each clinic, and to adjust the sample for equal weighting of each clinic. The proportion of participants from each clinic is 20% after adjusting for the weights. Descriptive analyses comparing proportions of participants by the measures described above were calculated using F‐tests to incorporate survey weights. Models were estimated using robust standard errors. All analyses were done using Stata 17.0 (Stata Statistical Software: Release 18 [computer program] 2022).

### 2.6 Sensitivity and Exploratory Analyses

We conducted several sensitivity and exploratory analyses to account for (1) different thresholds defining “high” stress, (2) covariate selection, and (3) gender.

First, to identify whether our analysis of whether COVID‐19 infection was associated with stress levels ≥13 was sensitive to the specific threshold level, we also tested lower thresholds of 11 and 12 (Appendix A1).

Second, we tested whether our results were sensitive to including multiple other sets of confounds using six additional linear regression models. These models sequentially added: comorbid health conditions (obesity, diabetes, heart disease, hypertension, and asthma/COPD); social determinants of health (household composition, employment status, job loss, and food insecurity); healthcare access (regular tribal payment and primary care provider); and pandemic well‐being impact (social isolation, missed important events, and loss of a loved one to COVID‐19). A final model included all additional covariates simultaneously. Full results of these analyses are reported in Appendix A2.

Third, we conducted an extensive analysis of the role of gender. Gender presents a potentially important dimension of COVID‐19, as men have experienced higher mortality rates (Alwani et al. 2021), but women in the United States during the COVID‐19 pandemic experienced higher levels of stress and anxiety (Cholankeril et al. 2023, Peyer et al. 2024). Thus, we checked for gender differences in the relationships between COVID‐19 infection and stress in all primary analyses (Appendix A3).

## 3 Results

A total of 788 AI/AN urban adults enrolled in the CONCERTS survey, and between 700 and 730 participants had complete data pertaining to our main exposures, outcomes, and covariates. After inverse probability weighting was applied to the sample, the mean age was 43.6 years; 60% were female; 23% had a college degree or higher (**Table** **1**). The mean PSS‐4 score was 6.5 (SD = 0.2).

**TABLE 1:** Characteristics of Urban American Indian and Alaska Native Adults, January–May 2021 (*N* = 788).

Characteristic	*N*	%
Perceived stress scale‐4 (PSS‐4) score^a^	6.5 (3.5)	
Unweighted, *N* = 720		
Age in years^a^	43.6 (14.1)	
Unweighted, *N* = 764		
**Gender**		
Male	246	39.9%
Female	521	60.1%
**COVID‐19 infection (self‐reported)**		
No	643	84.8%
Yes	101	15.2%
**Received COVID‐19 vaccine**		
No	273	50.5%
Yes	456	49.5%
**Number of times tested for COVID‐19**		
Never	122	20.0%
1 time	127	20.0%
2 times	134	16.2%
3 or more times	358	43.8%
**Highest level of completed education**		
Less than high school/no diploma	61	7.8%
High school graduate or GED	389	51.8%
Associate/occupational/technical/vocational degree	132	17.7%
College degree or higher	194	22.7%
**Total household income (past 12 months)**		
<$15,000	175	23.8%
$15,000–$34,999	197	28.2%
$35,000–$59,999	171	24.5%
≥$60,000	215	23.5%
**Household composition**		
Just me	169	20.5%
Living with spouse, no children	135	16.5%
Family with 2 or more generations	378	49.4%
None of these	98	13.6%
**Essential worker status**		
No	223	25.4%
Yes	377	52.3%
Not currently working	176	22.2%
**Experienced job loss due to COVID‐19**		
No	593	78.2%
Yes	136	21.8%
**Hospitalized due to COVID‐19** ^b^		
No	86	92.1%
Yes	9	7.9%
**Comorbidities**		
Any comorbidity (obesity, diabetes, heart disease, hypertension, or asthma)	528	67.0%
Obesity	294	40.5%
Diabetes	181	22.0%
Heart disease	57	6.9%
High blood pressure/hypertension	293	34.4%
Asthma or COPD	158	21.1%
**Food insecurity**		
No	494	62.1%
Yes	256	37.9%

*Note*: *N* reflects unweighted frequency counts. Percentages are weighted using inverse probability weights and may not sum to 100 due to rounding.

Abbreviations: PSS‐4 = Perceived Stress Scale‐4 (range 0–16); COPD = chronic obstructive pulmonary disease.

^a^ Mean (SD) presented for continuous variables; unweighted *N* shown below.

^b^ Restricted to COVID‐19‐positive participants only (unweighted, *n* = 95).

### 3.1 COVID‐19 Infection and Stress

Among all participants, 15.2% were infected with COVID‐19 nearly 1 year after the pandemic started. Among the 86 COVID‐19‐positive participants who reported the date of their first positive test, the mean time between survey completion and positive test date was 133.5 days (SD = 84.0). The mean perceived stress was 7.5 (SD = 0.5) for those who were infected with COVID‐19 and 6.4 (SD = 0.2) for those who were not.

We estimated similar associations of COVID‐19 infection with perceived stress using univariate and multivariate models. With our univariate model, COVID‐19 infection was associated with *β* = 1.1 (95% CI: 0.2, 2.1) higher perceived stress. Adjusting our estimates for the covariates of gender, household income, education, and age, COVID‐19 infection was associated with *β* = 0.9 (95% CI: 0.1, 1.8) higher perceived stress (**Table** **2**, **Panel A**).

**TABLE 2:** Associations between perceived stress scale‐4 scores and COVID‐19 infection among Urban American Indian and Alaska Native Adults between January and May 2021.

	Univariate model (1)	Multivariate model (2)
**Panel A: Association between COVID‐19 infection and stress. β (95% CI)**		
COVID‐19 infection	1.1	0.9
	(0.2, 2.1)	(0.1, 1.8)
*N*	730	700
**Panel B: Relative risk of perceived stress levels ≥6 and COVID‐19 infection. RR (95% CI)**		
COVID‐19 infection	1.1	1.1
	(1.0, 1.4)	(0.9, 1.3)
*N*	730	700
**Panel C: Relative risk of perceived stress levels ≥13 and COVID‐19 infection. RR (95% CI)**		
COVID‐19 infection	3.0	3.2
	(1.2, 7.8)	(1.2, 8.5)
*N*	730	700

*Note*: Panel A presents adjusted mean differences from linear regressions. Panels B and C present the relative risk of achieving a specific perceived threshold from modified Poisson regressions. Model 1 only includes COVID‐19 infection as an explanatory variable. Model 2 includes age, gender, household income, and education. All models use inverse probability weights; unweighted *N* presented.

We found that COVID‐19 infection was positively associated with the risk of stress over both primary thresholds using our univariate model. Using that model, COVID‐19 was associated with RR = 1.1 (95% CI: 1.0, 1.4) (**Table** **2**, **Panel B**) of stress levels ≥6 and RR = 3.0 (95% CI: 1.2, 7.8) of stress ≥13 (**Table** **2**, **Panel C**).

However, adjusting for age, gender, household income, and education, the 95% confidence interval of our estimated risk of stress ≥6 included 1.0 (RR = 1.1, 95% CI: 0.9, 1.3). In our multivariate model, COVID‐19 infection was still associated with a higher risk of perceived stress ≥13 (RR = 3.2, 95% CI: 1.2, 8.5).

Among the 86 COVID‐19‐positive participants who reported the date of their first positive test, the mean time between survey completion and positive test date was 133.5 days (SD = 84.0; median 123.5 days, interquartile range 78–164, range 5–429). Most participants with a positive COVID‐19 test were surveyed well beyond the acute phase of illness: 8% were within 4 weeks of their first positive test, 24% between 4 and 12 weeks, and 68% more than 12 weeks out. Adjusted mean perceived stress did not differ significantly across these categories: relative to the acute phase, *β* = 1.1 (95% CI: −3.0, 5.2) for the ongoing symptomatic group and *β* = 2.2 (95% CI: −0.6, 4.9) for the post‐COVID‐19 group. We also found no significant association between stress and days since first positive test (*β* = 0.006, 95% CI: −0.006, 0.018).

Categorizing infected participants by clinical phase yielded the same conclusion: perceived stress did not differ significantly across acute, ongoing‐symptomatic, and post‐COVID‐19 groups, and was, if anything, numerically highest among those surveyed more than 12 weeks after infection

### 3.2 Sensitivity and Exploratory Analyses

#### 3.2.1 Alternative Definitions of “High” Stress

Our finding that COVID‐19 infection was associated with higher risk of high stress was robust to the specified threshold. We found COVID‐19 infection was associated with an RR = 2.4 (95% CI: 1.0, 5.5) of stress ≥11 and an RR = 3.2 (95% CI: 1.3, 8.0) of stress ≥12 when adjusting for age, gender, income, and education (Appendix A1).

#### 3.2.2 Covariate Selection

Results were robust across a comprehensive set of alternative covariate specifications (Appendix A2). The association between COVID‐19 infection and perceived stress remained positive and of similar magnitude after adjusting for comorbid health conditions (*β* = 0.8, 95% CI: −0.1, 1.7), social determinants of health including food insecurity and employment status (*β* = 0.8, 95% CI: −0.1, 1.6), healthcare access variables (*β* = 0.8, 95% CI: −0.0, 1.7), and pandemic wellbeing impacts including social isolation and bereavement (*β* = 1.0, 95% CI: 0.1, 1.8). In a model including all additional covariates simultaneously, the estimate was somewhat attenuated but remained in the same direction (*β* = 0.6, 95% CI: −0.2, 1.5). Although the point estimate for COVID‐19 infection remained positive in the fully adjusted model, the 95% confidence interval included zero (*β* = 0.6, 95% CI: −0.2, 1.5), reflecting loss of statistical significance at the conventional *α* = 0.05 level. This attenuation is expected given the inclusion of fifteen additional covariates in a sample of this size, which substantially reduces statistical power. The stability of the point estimate across specifications, ranging from 0.6 to 1.0 across all models, suggests that the association is not an artifact of a single omitted confounder, even though precision is reduced in the most heavily parameterized model.

Notably, food insecurity was independently associated with higher perceived stress across models (*β* = 1.3, 95% CI: 0.5, 2.1), as was having a primary care provider (*β* = 1.1, 95% CI: 0.5, 1.8).

#### 3.2.3 Gender

We found no evidence that gender played an important role in the relationship between COVID‐19 infection and stress (Appendix A3). Men and women had similar associations between infection and mean stress, as well as the risk of high stress by both primary thresholds of 6 and 13. We also found no role of gender when examining secondary stress thresholds of 11 and 12 (Appendix A1).

## 4 Discussion

The COVID‐19 pandemic exacerbated existing psychosocial stressors and introduced new ones such as economic displacement, loss of loved ones, and fear of infection (Barzilay et al. 2020). Growing evidence indicates COVID‐19 infection itself is an important stressor, particularly in severe cases (Janiri et al. 2021, Zandifar et al. 2020). Our primary finding is that COVID‐19 infection was associated with higher mean perceived stress in AI/AN patients from geographically dispersed healthcare organizations.

The robustness of our primary finding across multiple sensitivity analyses strengthens confidence in the observed association. The estimate for COVID‐19 infection and perceived stress was largely stable whether or not we adjusted for comorbid conditions, social determinants of health, healthcare access, or pandemic‐related wellbeing impacts, including social isolation and loss of a loved one to COVID‐19. Taken together, these sensitivity analyses suggest that the association between COVID‐19 infection and elevated perceived stress is unlikely to be fully explained by the comorbid conditions, social disadvantages, or pandemic‐related stressors that disproportionately affect urban AI/AN populations.

The practical significance of these associations is likely more important on a population, rather than individual, level. On the continuous PSS‐4 scale, the adjusted 0.9‐point difference associated with COVID‐19 infection corresponds to a standardized effect of roughly 0.26 standard deviations (weighted SD = 3.5), below the half‐standard‐deviation change commonly cited as a threshold for individual‐level clinical importance. At the individual level, then, the average shift in perceived stress is modest. The more clinically salient consequence emerges at the population level: because a small upward shift in a population's mean can substantially enlarge the proportion in the high‐stress tail, COVID‐19 infection was associated with a near‐tripling of the risk of very high stress (PSS‐4 ≥ 13). One study by Redmond et al. found that individuals with high PSS‐4 scores had an elevated risk of incident total coronary heart disease compared with those with no stress (Redmond et al. 2013). Although we used different thresholds to define high stress than Redmond et al., who set thresholds at 1, 3, and 5, we observed that COVID‐19 infection was associated with a higher risk of individuals reporting stress scores ≥13, indicating that risks like coronary heart disease associated with high stress scores may be a concern in AI/AN people who were infected with COVID‐19. That said, we do note that the PSS‐4 has no formally established minimal clinically important difference.

Although this study does not demonstrate a causal link between contracting COVID‐19 and stress, below, we explore potential mechanisms by which COVID‐19 might increase stress, and also consider the potential for the inverse causal relationship, or other confounds.

Several potential nonexclusive biological and psychosocial mechanisms may link infection and stress. On a biological level, while the etiology of COVID‐19‐related psychoneurological damage is not fully understood, the virus's neurotropic properties suggest direct effects on the central nervous system (Nalbandian et al. 2021, Thye et al. 2022). Systemic inflammation associated with COVID‐19 can contribute to neuropsychiatric symptoms through activation of the hypothalamic–pituitary–adrenal (HPA) axis and subsequent dysregulation of stress hormones. Beyond the HPA axis, emerging evidence points to disruption of serotonin and dopamine signaling via persistent neuroinflammation and oxidative stress (OA et al. 2025). On a psychosocial level, COVID‐19 infection may lead to deleterious socioeconomic effects, such as job loss or economic hardship, outcomes demonstrated in this sample in another study (Henderson et al. 2025) that may increase stress, though adjusting our results for job loss and food insecurity did not affect our estimates of the association between COVID‐19 infection and stress. Additionally, the experience of an unanticipated COVID‐19 infection may be a particularly salient event that is well captured by the PSS‐4, which specifically elicits feelings about the uncontrollability and unpredictability of life.

Importantly, there are also plausible pathways running in the opposite direction, which our cross‐sectional design cannot rule out. It is well‐established that chronic stress can suppress immune function (Segerstrom and Miller 2004), potentially rendering individuals with high baseline stress more susceptible to contracting or experiencing a more severe course of COVID‐19. Furthermore, high‐stress environments, such as high‐risk essential work, may simultaneously increase both perceived stress and the likelihood of viral exposure, although we did not find that adjusting for essential work status meaningfully changed our estimated associations. Recent prospective evidence is consistent with this reverse pathway: a 2025 cohort study found that higher perceived chronic stress before COVID‐19 infection, measured by the PSS‐10 in the week following a positive COVID‐19 test, independently predicted persistence of symptoms at 1 month (Fazekas et al. 2026). Although that study examined symptom persistence rather than initial infection risk, it illustrates how pre‐existing stress and COVID‐19 outcomes are bidirectionally entangled in ways that cross‐sectional studies cannot fully disentangle.

A key concern in cross‐sectional studies of this kind is whether any observed association reflects a persistent effect of infection or merely captures acute stress proximate to the illness episode. To explore this, we examined whether time elapsed since first positive COVID‐19 test moderated the association with perceived stress. In adjusted models restricted to COVID‐19‐positive participants, days since first positive test was not a significant continuous predictor of stress. Categorizing infected participants by clinical phase (acute, ≤4 weeks; ongoing symptomatic, >4–12 weeks; post‐COVID‐19, >12 weeks) produced the same pattern, with no significant gradient and the highest stress among those furthest from infection, an observation difficult to reconcile with the alternative that the association merely reflects transient distress in the immediate aftermath of illness. While statistical power is limited by the relatively small number of infected participants with a valid test date, these findings are at least consistent with the possibility that stress associated with COVID‐19 infection may persist beyond the acute phase of illness, a pattern observed in studies of long COVID and postinfectious psychological sequelae. Longitudinal studies tracking COVID‐19 survivors across multiple time points have found that mental health impairments, including anxiety and depression, persist at 6 and 12 months postinfection, with younger adults and those with more severe acute illness at greatest risk (Badinlou et al. 2024). Furthermore, a study of COVID‐19 survivors assessed approximately two years postillness found that symptoms of anxiety, depression, and perceived stress remained prevalent, with women exhibiting particularly elevated stress levels (Rivas Nieto et al. 2026). The 10‐item PSS was used in a Hong Kong study of severe acute respiratory syndrome survivors and matched community controls (Lee et al. 2007). Survivors had higher stress levels during and after the outbreak compared with controls; this gap in stress levels persisted 1 year after the outbreak.

The implications of these findings are particularly salient for AI/AN communities, where the literature on stress and COVID‐19 remains limited. Only a few studies of stress and COVID‐19 infection have included AI/AN people; none that we are aware of examined the association between COVID‐19 infection and perceived stress in individuals. In a nationally representative online survey of 5500 Americans conducted from December 2020 to February 2021, discrimination, a chronic form of stress, was examined with an adapted Everyday Discrimination Scale. AI/AN adults had higher odds than non‐Hispanic White adults, and similar odds to Asian adults, of experiencing COVID‐19‐related discrimination (Strassle et al. 2022). A 2020 survey of 167 adults residing on the Blackfeet reservation examined perceived control over contracting COVID‐19, ability to cope with pandemic stressors, psychological stress linked to the pandemic, and sleep health. Perceived greater control over contracting the virus was linked to better sleep health (John‐Henderson et al. 2021). Our study thus provides a link between the COVID‐19 pandemic and stress in AI/AN peoples that identifies individual‐level risk factors for elevated stress.

In addition to the concerns related to coronary heart disease risk raised above, elevated long‐term stress is concerning because perceived stress has been associated with other negative health outcomes in AI and First Nations peoples. In a small sample (*N* = 44) of Hopi female caregivers, mean stress as measured by the PSS‐10 was associated with reported poor health (Cordova‐Marks et al. 2020). In a study of 684 pregnant women in Manitoba, 37% of whom were Aboriginal, high stress as defined by a PSS‐4 score of ≥11 was associated with elevated risk of preterm birth (Heaman et al. 2005). Notably, this study defined high stress as greater than or equal to 2 standard deviations above the mean, which also corresponds to the cutoff of 13 used in our primary analysis, and we also determined in our sensitivity analysis that COVID‐19 infection was associated with a higher risk of a PSS‐4 score of ≥11. A 2021 study found that elevated perceived stress was associated with greater risk of diabetes and prediabetes in an AI sample (Nikolaus et al. 2021). Lewis et al. in their 2021 work proposed a stress‐cardiovascular disease pathway that could explain some cardiovascular disease disparities between Indigenous peoples and other populations (Lewis et al. 2021). Long‐term stress has been linked to cardiovascular disease (Redmond et al. 2013) and diabetes (Hackett and Steptoe 2017) in other populations.

More research is warranted into the relationship between stress and health in AI/AN peoples. Future research should also examine how COVID‐19‐related stress may differentially impact high‐risk subgroups, including individuals with pre‐existing cardiometabolic conditions, older adults, those in high‐risk occupations such as healthcare workers, and communities facing adverse socioeconomic conditions, as the health consequences of elevated stress are likely to be magnified in these populations.

### 4.1 Limitations

We acknowledge several study limitations. First, all data were self‐reported, including our main variables of stress and COVID‐19 infection. The reliability of self‐reported COVID‐19 infection has been questioned, at least among essential workers (Mulchandani et al. 2021, Maharaj et al. 2022). Furthermore, our reliance on self‐reported COVID‐19 infection introduces the potential for recall bias, specifically a form of differential recall influenced by a participant's current psychological state (Kip et al. 2001). Because perceived stress was measured at the same time as the retrospective reporting of past infection, individuals experiencing higher levels of current distress may have been more prone to recall or attribute prior respiratory illnesses specifically to COVID‐19. This cognitive tendency, where current psychological symptoms can color the memory and interpretation of past health events, could lead to an overestimation of the association between COVID‐19 infection and current stress levels. While we attempted to mitigate this by requiring participants to report specific “external” markers, such as a positive test result or a professional diagnosis, rather than symptoms alone, the lack of clinical confirmation via laboratory‐verified records remains a limitation. Furthermore, our reliance on self‐reported diagnosis likely missed individuals with asymptomatic infections who were never tested. This form of misclassification bias, where truly infected but asymptomatic individuals are grouped with the ‘uninfected’ reference group, generally leads to an underestimation of the true association. If asymptomatic infection contributes to psychological or physiological stress, its presence in our control group would dampen the observed difference between groups. Relatedly, we do not have data to determine if patients were tested for appropriate reasons or whether they had social, logistical, or clinical contraindications.

Second, our measure of time since COVID‐19 infection was derived from participants' self‐reported date of first positive test, which introduces two notable limitations. First, as with our primary infection measure, this date relies on self‐report and is subject to recall bias. Second, and more importantly, it captures only the first recorded infection, meaning that for participants who experienced multiple COVID‐19 infections, this variable does not reflect the most recent or necessarily the most clinically relevant episode. As a result, our analysis of time since infection should be interpreted with caution, and the absence of a significant association between days since first positive COVID‐19 test and stress may partly reflect measurement error in this variable rather than a true null relationship.

Third, our study participants were seen at healthcare organizations that primarily serve AI/AN people in urban settings; however, some patients may have traveled from rural areas to receive care. Moreover, findings from urban settings may not generalize to AI/AN populations living in rural settings and reservations.

Fourth, our response rate (17.1%) was low, with unknown selection biases, although this response rate was similar to that of a 2021 study of COVID‐19‐related discrimination in diverse populations (Strassle et al. 2022). Survey respondents may differ substantially from nonrespondents, limiting generalizability to the latter and to whole populations, even with our demographic‐based survey weights. More specifically, nonresponse bias could affect not only the generalizability of our descriptive estimates but also the internal validity of the estimated association, if the propensity to respond was jointly related to both COVID‐19 infection and perceived stress. We have no strong a priori reason to expect such joint dependence to operate in a particular direction, and our inverse‐probability weights, which correct for nonresponse by age and gender, partially mitigate this concern; however, weighting cannot adjust for unmeasured determinants of nonresponse, so residual nonresponse bias of uncertain direction cannot be excluded. For instance, the survey may have disproportionately attracted individuals with more discretionary time, those with a greater need for the survey incentive, those with higher health consciousness, or conversely, those experiencing greater pandemic‐related distress who felt more motivated to share their experiences. These unknown selection biases limit the generalizability of our findings. Relatedly, the $100 incentive provided to participants, while consistent with the level of compensation we have found necessary to achieve meaningful response rates in AI/AN populations, may itself have introduced selection bias by disproportionately attracting individuals for whom this amount was a significant financial motivator. To the extent that financial strain is independently associated with perceived stress, this selection mechanism could inflate mean stress levels in our sample relative to the broader patient population, though the direction of any resulting bias on our estimated association between COVID‐19 infection and stress is less clear.

Fifth, we were unable to account for two factors that may shape the association between COVID‐19 infection and perceived stress: the clinical severity of infection and pre‐existing mental health conditions. Although participants reported whether they had been hospitalized for COVID‐19, only nine infected participants had been hospitalized, too few to meaningfully analyze severity (among them, mean perceived stress was numerically higher, but the small number precludes inference). More severe infection has been associated with greater psychological sequelae, including posttraumatic stress and depression, so the absence of severity data is a limitation. Our survey also did not capture pre‐existing mental health diagnoses such as anxiety or depression, which could plausibly influence both the likelihood of attributing distress to infection and current perceived stress; we were therefore unable to adjust for this potential confounder.

Sixth, survey fatigue may have influenced our findings, as long surveys can lead participants to provide less accurate or repetitive responses toward the end of a survey to minimize cognitive burden (Krosnick 1991). As the CONCERTS survey required approximately 40 min to complete, this may have influenced the data quality of measures located in the latter sections. However, we have several reasons to believe the impact on our primary outcome was mitigated. First, the PSS‐4 and COVID‐19 infection questions were roughly halfway through the survey. Second, a post hoc analysis of internal consistency for the PSS‐4 demonstrated a Cronbach's alpha of 0.7, suggesting that participants remained sufficiently attentive to these items. Nonetheless, survey fatigue may have contributed to our lower overall response rate and may result in an under‐ or overestimation of associations.

Seventh, estimates of the relative risk of very high perceived stress (PSS‐4 ≥13) should be interpreted with caution, as the small number of participants meeting this threshold results in wide confidence intervals and considerable uncertainty around the magnitude of the association.

Finally, the cross‐sectional design of this study presents a primary limitation by precluding the determination of causality.

## 5 Conclusion

COVID‐19 infection was significantly associated with elevated perceived stress among urban AI/AN patients at 5 geographically dispersed healthcare organizations. The association of COVID‐19 infection with perceived stress did not depend on the threshold used to define high stress, was robust to multiple sets of tested covariates, and did not depend upon the gender of the participant. Healthcare providers should be aware that AI/AN people who have been infected with COVID‐19 are at higher risk for elevated stress, and future research is warranted into the long‐term associations between COVID‐19 infection and stress and potential health consequences, particularly in AI/AN communities where the burden of both COVID‐19 and stress‐related comorbidities has been disproportionately high.

## Author Contributions

**Austin Henderson**: Conceptualization, methodology, data curation, formal analysis, software, writing – original draft, writing – review and editing, funding acquisition. **Richard MacLehose**: Investigation, methodology, writing – review and editing, project administration, funding acquisition. **Spero M. Manson**: Investigation, writing – review and editing, project administration, funding acquisition. **Dedra Buchwald**: Investigation, writing – review and editing, project administration, funding acquisition, supervision.

## Funding

This work was funded by the National Institutes of Health (U54MD011240‐05S1, Contact Principal Investigator, D. Buchwald).

## Ethics Statement

This study was approved by the Washington State University Institutional Review Board (#18590), the Alaska Area Institutional Review Board (#2020‐11‐044), and Tribal research review committees at Southcentral Foundation (SCF) and the Alaska Native Tribal Health Consortium. Tribal approval was also obtained before dissemination.

## Conflicts of Interest

The authors declare no conflicts of interest.

## Supporting information




**Supporting File 1**: brb371783‐sup‐0001‐SuppMat.pdf

## Data Availability

The data are available from the corresponding author upon reasonable request.

## Appendix A1 Sensitivity Analysis: Different Thresholds of High Stress

Because the perceived stress scale is not a diagnostic instrument, thresholds for different “high” levels of stress are somewhat arbitrary and often practically determined by sample characteristics such as mean and median. While we chose thresholds of (1) 6 and (2) 13 in our primary analysis because they correspond to (1) the mean and (2) the highest quartile of possible scores, there are multiple other thresholds that could have reasonably been chosen. We present our estimates of the relative risk of “high” stress, defined by a threshold of 11 in Appendix Table A1 and a treshold of 12 in Appendix Table A2, associated with COVID‐19 infection. Additionally, we conducted an analysis of gender to see if these results were meaningfully dependent upon the gender of the participant.

In all cases, we found our estimates were not meaningfully sensitive to the threshold chosen or gender, indicating that we can be more confident that COVID‐19 infection is in fact associated with high stress, however defined.

**TABLE A1:** Perceived stress threshold of 11.

	Both genders (1)	Men (2)	Women (3)	Interaction (4)
**COVID‐19 infection**	2.0	2.3	1.9	2.4
	(1.1, 3.8)	(0.7, 7.3)	(0.9, 4.0)	(0.7, 7.5)
**Female (vs. male)**	1.2			1.3
	(0.6, 2.3)			(0.6, 2.6)
**Female * COVID‐19 infection**				*p* = 0.77
***N***	689	221	468	689

*Note*: Association between very high (≥11) perceived stress scores and COVID‐19 infection. Estimates are relative risks from a modified Poisson regression. Model 1 includes data from both genders with gender as a control variable. Models 2 and 3 stratify by gender, with Model 2 using data from men only and Model 3 using data from women only. Model 4 uses data from both genders with an added interaction term between gender and COVID‐19 infection. All estimates adjusted for age, gender, household income, education, and site. 95% Confidence interval in parentheses, with only the *p*‐value displayed for the interaction term in Model 4.

**TABLE A2:** Perceived stress threshold of 12.

	Both genders (1)	Men (2)	Women (3)	Interaction (4)
**COVID‐19 infection**	2.7	3.4	2.4	2.9
	(1.3, 5.8)	(1.1, 10.9)	(0.9, 6.4)	(0.9, 9.4)
**Female (vs. male)**	0.7			0.7
	(0.3, 1.5)			(0.3, 1.7)
**Female * COVID‐19 infection**				*p* = 0.89
**N**	689	221	468	689

*Note*: Association between very high (≥12) perceived stress scores and COVID‐19 infection. Estimates are relative risks from a modified Poisson regression. Model 1 includes data from both genders with gender as a control variable. Models 2 and 3 stratify by gender, with Model 2 using data from men only and Model 3 using data from women only. Model 4 uses data from both genders with an added interaction term between gender and COVID‐19 infection. All estimates adjusted for age, gender, household income, education, and site. 95% Confidence interval in parentheses, with only the *p‐*value displayed for the interaction term in Model 4.

## Appendix A2 Sensitivity Analysis: Covariate Selection

We examined whether the covariates of living in a multigenerational household or experiencing job loss, both of which are plausibly related to both COVID‐19 infection and stress, could potentially moderate our primary estimates. We present variable definitions below, and then report estimates in **Appendix Table** **A3**.

**Age**. Age was measured in years as a continuous variable.

**Gender**. Participants were asked, “What term best expresses how you describe your gender identity?” For analysis, responses were collapsed into male and female categories. Nonbinary, transgender, agender, bigender, and other gender identities were excluded from the primary analysis due to small cell sizes.

**Education**. Participants were asked about the highest level of education they had completed. Response options were: less than high school/no diploma, high school graduate or GED, some college, associate/occupational/technical/vocational degree, and college degree or higher. For analysis, “some college” was collapsed with the associate degree category.

**Household income**. Participants reported their total household income in the past 12 months, including wages, Social Security, disability, and child support, using 15 ordered categories ranging from under $10,000 to more than $150,000. For analysis, responses were collapsed into four categories: <$15,000, $15,000–$34,999, $35,000–$59,999, and ≥$60,000.

**Household composition**. Household composition was determined by the response to the question, “What best describes your family at home?” Response options were: just me, living with spouse/no kids, family including kids, family with 3 generations (parents, children, grandchildren), family with 4 generations, and none of these. For analysis, responses were collapsed into “Just me,” “Living with spouse, no children,” “Family with two or more generations” (combining the family‐including‐kids, 3‐generation, and 4‐generation categories), and “None of these.”

**Job loss**. Job loss was assessed through the question, “Have you experienced any of the following difficulties due to the COVID‐19 pandemic?” The relevant sub‐item asked participants to indicate Yes or No for “Losing my job.”

**Comorbidities**. The presence of specific health conditions was assessed by asking, “Have you ever been told by a doctor, nurse, or other health care professional that you have any of the following health conditions?” Conditions assessed included obesity, diabetes, heart disease (e.g., congestive heart failure or heart attack), high blood pressure, and asthma or COPD. Each condition was coded as Yes (1) or No (0) and entered as separate binary covariates in the relevant model.

**Essential worker status**. Participants were asked, “Are you considered an essential worker? An essential worker is someone who was required to go to work even when stay‐at‐home orders were in place.” Response options were Yes, No, and Not currently working.

**Food insecurity**. Food insecurity was assessed using six items adapted from the USDA Household Food Security Survey Module. Participants were asked how often each of the following was true in the past 12 months: (1) the food their household bought did not last and there was not enough money to get more (often true/sometimes true/never true); (2) their household could not afford to eat balanced meals (often true/sometimes true/never true); and whether they or anyone in their household (3) cut down meal sizes or skipped meals due to insufficient money for food (yes/no); (4) ate less than they felt they should because of insufficient money (yes/no); and (5) were hungry but did not eat because they could not afford enough food (yes/no). Participants who answered affirmatively to any of these five items were classified as food insecure (yes); all others were classified as food secure (no).

**Regular tribal payment**. Participants were asked, “Do you receive any regular form of payment from your tribe, for example, dividends from a native corporation or per capita cash transfer?” Responses were coded as yes (1) or no (0).

**Primary care provider**. Participants were asked, “Do you have a primary care provider or doctor, nurse, or other health care professional you see regularly?” Responses were coded as yes (1) or no (0).

**Social isolation**. Social isolation was assessed through the question, “Have you experienced any of the following difficulties due to the COVID‐19 pandemic?” The relevant sub‐item asked participants to indicate yes or no for “Being isolated from others who are important to me.”

**Missed important events**. Assessed through the same COVID‐19 pandemic difficulties question, with the relevant sub‐item: “Missing important events (for example, funeral, ceremony, spiritual or religious activities).” Responses were coded as yes (1) or no (0).

**Lost a loved one to COVID‐19**. Assessed through the same COVID‐19 pandemic difficulties question, with the relevant sub‐item: “Losing a loved one to COVID‐19 disease.” Responses were coded as yes (1) or no (0).

**TABLE A3:** Sensitivity analysis: associations between COVID‐19 infection and perceived stress (PSS‐4) across alternative covariate specifications.

	(2) Household composition	(3) Job loss	(4) Comorbidities	(5) Social det. of health	(6) Healthcare access	(7) Pandemic wellbeing	(8) Full model
**COVID‐19 Infection (ref. = No)**							
Yes	0.9 (0.0, 1.8)	0.8 (−0.0, 1.7)	0.9 (0.0, 1.8)	0.8 (−0.1, 1.7)	0.8 (−0.1, 1.6)	0.8 (−0.0, 1.7)	1.0 (0.1, 1.8)
**Gender (ref. = male)**							
Female	0.3 (−0.3, 1.0)	0.4 (−0.3, 1.0)	0.3 (−0.3, 1.0)	0.2 (−0.5, 0.8)	0.3 (−0.4, 1.0)	0.3 (−0.3, 0.9)	0.3 (−0.3, 0.9)
**Household income (ref. = <$15,000)**							
$15,000–$34,999	−1.7 (−2.4, −0.9)	−1.6 (−2.4, −0.8)	−1.6 (−2.4, −0.8)	−1.6 (−2.4, −0.8)	−1.4 (−2.2, −0.5)	−1.7 (−2.5, −0.9)	−1.8 (−2.6, −1.0)
$35,000–$59,999	−1.8 (−2.7, −0.9)	−1.9 (−2.8, −1.0)	−1.8 (−2.7, −0.9)	−1.8 (−2.7, −0.9)	−1.4 (−2.3, −0.4)	−1.8 (−2.7, −0.9)	−1.9 (−2.8, −1.1)
≥$60,000	−2.9 (−3.8, −1.9)	−2.9 (−4.0, −1.9)	−2.8 (−3.8, −1.8)	−3.0 (−3.9, −2.0)	−2.1 (−3.3, −1.0)	−2.9 (−3.9, −2.0)	−3.0 (−4.0, −2.0)
**Education (ref. = less than high school)**							
High school/GED	−0.6 (−1.8, 0.5)	−0.7 (−1.8, 0.5)	−0.6 (−1.7, 0.5)	−0.3 (−1.3, 0.7)	−0.8 (−1.9, 0.4)	−0.6 (−1.6, 0.5)	−0.5 (−1.6, 0.7)
Associate/vocational degree	0.1 (−1.2, 1.5)	0.1 (−1.3, 1.4)	0.1 (−1.3, 1.5)	0.4 (−0.8, 1.7)	−0.2 (−1.6, 1.2)	0.2 (−1.2, 1.5)	0.2 (−1.2, 1.6)
College degree or higher	−0.2 (−1.3, 1.0)	−0.3 (−1.5, 1.0)	−0.1 (−1.3, 1.1)	0.3 (−0.7, 1.4)	−0.3 (−1.6, 0.9)	−0.2 (−1.3, 1.0)	−0.0 (−1.3, 1.2)
**Age (ref. = 18–29)**							
30–39	0.2 (−0.6, 1.1)	0.2 (−0.7, 1.1)	0.2 (−0.7, 1.1)	0.1 (−0.8, 1.0)	−0.0 (−0.9, 0.8)	0.2 (−0.6, 1.1)	0.1 (−0.8, 1.0)
40–49	−0.3 (−1.2, 0.6)	−0.3 (−1.2, 0.6)	−0.2 (−1.1, 0.7)	−0.6 (−1.5, 0.3)	−0.2 (−1.2, 0.7)	−0.4 (−1.3, 0.4)	−0.4 (−1.3, 0.5)
50–59	−0.8 (−1.9, 0.3)	−1.0 (−2.0, 0.1)	−0.8 (−1.9, 0.3)	−1.1 (−2.1, −0.0)	−1.0 (−2.0, 0.1)	−1.0 (−2.1, −0.0)	−1.0 (−2.0, 0.1)
60–69	−1.2 (−2.1, −0.2)	−1.3 (−2.3, −0.4)	−1.2 (−2.1, −0.2)	−0.8 (−1.8, 0.1)	−1.4 (−2.4, −0.4)	−1.2 (−2.2, −0.3)	−1.2 (−2.1, −0.3)
70+	−2.7 (−3.9, −1.6)	−3.0 (−4.2, −1.8)	−2.6 (−3.8, −1.4)	−2.8 (−4.1, −1.5)	−2.8 (−3.9, −1.6)	−3.0 (−4.1, −1.8)	−2.8 (−4.0, −1.7)
**Additional covariates (selected models only)**							
Household composition (ref. = just me)							
Living with spouse, no children	—	0.4 (−0.6, 1.4)	—	—	0.4 (−0.6, 1.4)	—	—
Family with 2+ generations	—	−0.1 (−0.9, 0.8)	—	—	−0.1 (−1.0, 0.7)	—	—
None of these	—	−0.9 (−1.9, 0.2)	—	—	−0.7 (−1.7, 0.4)	—	—
Job loss: Yes (ref. = No)	—	—	0.0 (−0.7, 0.7)	—	−0.1 (−0.8, 0.6)	—	—
Obesity: Yes (ref. = No)	—	—	—	0.8 (0.1, 1.5)	—	—	—
Diabetes: Yes	—	—	—	0.2 (−0.7, 1.1)	—	—	—
Heart disease: Yes	—	—	—	−0.4 (−1.7, 1.0)	—	—	—
High blood pressure: Yes	—	—	—	−0.3 (−1.0, 0.4)	—	—	—
Asthma/COPD: Yes	—	—	—	0.5 (−0.3, 1.2)	—	—	—
Essential worker: Yes (ref. = No)	—	—	—	—	0.3 (−0.4, 1.0)	—	—
Not currently working	—	—	—	—	0.3 (−0.5, 1.2)	—	—
Any food insecurity: Yes (ref. = No)	—	—	—	—	1.3 (0.5, 2.1)	—	—
Regular tribal payment: Yes (ref. = No)	—	—	—	—	—	−0.3 (−0.9, 0.3)	—
Has primary care provider: Yes (ref. = No)	—	—	—	—	—	1.1 (0.5, 1.8)	—
Social isolation: Yes (ref. = No)	—	—	—	—	—	—	0.4 (−0.4, 1.3)
Missed important events: Yes (ref. = No)	—	—	—	—	—	—	0.1 (−0.6, 0.8)
Lost loved one to COVID‐19: Yes (ref. = No)	—	—	—	—	—	—	0.1 (−0.6, 0.8)

*Note*: β coefficients (95% confidence intervals) from linear regression models with PSS‐4 total score as the outcome. All models include inverse probability weights and site fixed effects. Model 1 replicates the primary multivariate model. Models 2–3 add household composition and job loss, respectively (original Appendix A.2 models). Model 4 adds comorbid conditions (obesity, diabetes, heart disease, hypertension, asthma/COPD). Model 5 adds social determinants of health (household composition, employment status, job loss, food insecurity). Model 6 adds healthcare access variables (regular tribal payment, primary care provider). Model 7 adds pandemic well‐being impact variables (social isolation, missed events, loss of loved one). Model 8 includes all additional covariates simultaneously. — = variable not included in this model. PSS‐4 = Perceived Stress Scale‐4; COPD = chronic obstructive pulmonary disease.

## Appendix A3 Sensitivity Analysis: Gender

To examine the role of gender in the relationship between COVID‐19 infection and stress, we took several approaches, with results presented in Appendix Table A4 and Appendix Table A5. The first regression includes gender as an adjustment variable (along with household income, education, and age); the second and third regressions stratify the analysis by gender and are run once for men and once for women. The fourth includes adjustment variables listed above and an interaction term between gender and COVID‐19 infection. We took the same approach with the threshold analysis, in which we first included gender as a control variable, then stratified by gender in the second and third regressions, and examined the interaction of gender with the primary exposure variable in the fourth regression.

Mean stress scores were 6.2 (SD = 0.3) for men and a mean score of 6.8 (SD = 0.2) for women. Stratifying by gender, we found higher stress levels in both men and women who were infected with COVID‐19. Women who were infected with COVID‐19 had a mean perceived stress of 7.7 (SD = 0.6); uninfected women had a mean score of 6.6 (SD = 0.6). Men who were infected with COVID‐19 had a mean perceived stress of 7.0 (SD = 0.7), while uninfected men had a mean score of 6.1 (SD = 0.3).

We found similar associations between reported COVID‐19 infection and perceived stress in men and women, with *β* = 1.0 (95% CI: −0.3, 2.4) in men and β = 0.7 (95% CI: −0.4, 1.8) in women. In the fourth model, which used pooled gender data and an interaction term between stress and gender, that interaction was not significant (*p* = 0.68). Relatedly, we found no gender differences in how COVID‐19 infection was associated with stress levels ≥6 or ≥13.

**TABLE A4:** Characteristics of the CONCERTS study sample by gender, January–May 2021 (*n* = 767 with nonmissing gender data).^a^

	Male *N* = 246 (39.9%)		Female *N* = 521 (60.1%)		Total *N* = 767	
Perceived Stress Scale 4 Score (Hammen et al. 2009)	6.2	(3.5)	6.8	(3.4)	6.5	(3.4)
Age in years (Hammen et al. 2009)	43.2	(13.6)	43.9	(14.4)	43.6	(14.1)
Tested positive for or told by health professional that have COVID‐19		15.1%		14.9%		14.9%
Received COVID‐19 vaccine		50.3%		49.0%		49.5%
Number of times tested for COVID‐19						
Never		19.3%		20.7%		20.1%
1 time		25.7%		16.6%		20.2%
2 times		16.7%		15.6%		16.0%
3 or more times		38.4%		47.0%		43.6%
Highest level of completed education						
Less than high school, no diploma		5.7%		9.2%		7.8%
High school graduate or GED		55.8%		48.9%		51.7%
Associate, occupational, technical, or vocational degree		18.4%		17.2%		17.7%
College degree or higher		20.1%		24.7%		22.9%
Total household income past 12 months						
<$15,000		21.1%		25.3%		23.6%
$15,000–34,999		25.0%		30.6%		28.3%
$35,000–59,999		28.1%		22.2%		24.6%
***≥*** $60,000		25.8%		21.9%		23.4%

*Note*: N = 767 reflects participants with nonmissing data on gender. The 21 participants excluded from this table did not report a binary gender identity and are included in all nongender‐stratified analyses.

^a^ Mean (SD) displayed for PSS‐4 score and age.

**TABLE A5:** Associations between perceived stress and COVID‐19 infection and stress with a focus on the role of gender.

	Both genders (1)	Men (2)	Women (3)	Interaction (4)
**Panel A: Association between COVID‐19 infection and stress. β (95% CI)**				
COVID‐19 infection	0.9	1.0	0.7	1.1
	(0.1, 1.8)	(−0.3, 2.4)	(−0.4, 1.8)	(−0.3, 2.5)
				
Female (vs. male)	0.3			0.4
	(−0.3, 1.0)			(−0.3, 1.1)
				
Female * COVID‐19 infection				*p* = 0.68
*N*	691	222	469	691
**Panel B: Relative risk of perceived stress levels ≥6 and COVID‐19 infection. RR (95% CI)**				
COVID‐19 infection	1.1	1.1	1.1	1.2
	(0.9, 1.3)	(0.8, 1.5)	(0.9, 1.3)	(0.8, 1.6)
	1.1			1.1
Female (vs. male)	(0.9, 1.3)			(0.9, 1.3)
	1.1			1.1
				
Female * COVID‐19 infection				*p* = 0.69
*N*	691	222	469	691
**Panel C: Relative risk of perceived stress levels ≥13 and COVID‐19 infection. RR (95% CI)**				
COVID‐19 infection	3.2	2.3	3.3	2.0
	(1.2, 8.5)	(0.5, 10.8)	(0.8, 13.6)	(0.4, 10.6)
				
Female (vs. male)	0.5			0.4
	(0.2, 1.5)			(0.1, 1.4)
				
Female * COVID‐19 infection				*p* = 0.43
N	691	222	469	691

*Note*: Panel A presents adjusted mean differences from linear regressions; Panels B and C present relative risk from modified Poisson regressions. In all cases, Model 1 includes data from both genders with gender as a control variable; Models 2 and 3 stratify by gender, with Model 2 using data from men only and Model 3 using data from women only; Model 4 uses data from both genders with an added interaction term between gender and COVID‐19 infection. 95% Confidence interval in parentheses, with only the *p*‐value displayed for the interaction term in Model 4.
