# Supplementary material for: COVID‐19 Infection Is Associated With Higher Stress in Urban American Indian and Alaska Native Peoples: Results From a Cross‐Sectional Survey
Source: Brain Behav. 2026 Sep 17;16(9):e71783. doi: 10.1002/brb3.71783 (PMC13586663; doi:10.1002/brb3.71783)
Supplement: Supplementary file 1 [file BRB3-16-e71783-s001.pdf]

Codebook

Data Dictionary Codebook

12/19/2021 1:12pm

Collapse all instruments

#	Variable / Field Name	Field Label<br>*Field Note*	Field Attributes (Field Type, Validation, Choices, Calculations, etc.)
Instrument: **CONCERTS Study Info and Consent** (concerts_study_info_and_consent) Collapse			
1	record_id	Record ID	text
2	today	Today's date	text (date_mdy)<br>Field Annotation: @TODAY
3	age	How old are you?<br>*years old*	text (integer)
4	aian	Are you American Indian or Alaska Native?	yesno<br>1 Yes<br>0 No

5	consent<br>Show the field ONLY if:<br>[age] >= 18 and [aian] = '1'	WASHINGTON STATE UNIVERSITYResearch Study Consent FormElson S. Floyd College of Medicine, Community HealthStudy Title: Community Organizations for Natives: COVID-19 Epidemiology, Research, Testing, and Services (CONCERTS)Researcher: Dedra Buchwald, MD, Principal Investigator, 206-883-4740Sponsor: National Institutes of HealthContact information: Tara Nolen, tara.nolen@hunterhealth.org, (316) 491-7692 ext 1170You are being asked to take part in a research study carried out by Dedra Buchwald, MD. This form explains the research study and your part in it if you decide to join the study. Please read the form carefully, taking as much time as you need. Ask the researcher to explain anything you do not understand. Your participation in the study is voluntary. You can decide not to join the study. If you join the study, you can change your mind later or quit at any time. You may refuse any question, test, or procedure. There will be no penalty or loss of services or benefits if you decide to not take part in the study or quit later. We will contact you if we have any questions about your answers or need clarification.What is the purpose of this study? To find ways to improve COVID-19 testing for American Indian and Alaska Native people. You cannot take part in this study if you are not American Indian or Alaska Native.What will I be asked to do if I am in this study?You will be asked to complete a survey about your health and well-being, as well as your thoughts, attitudes, and feelings about services for COVID-19 testing. The survey will take up to 40 minutes to complete. Are there any benefits to me if I am in this study?You may help your local clinic improve testing for COVID-19 for American Indian and Alaska Native people.Are there any risks to me if I am in this study?You may be uncomfortable answering some of the survey questions. You can decide to skip any question that you wish. You can also stop taking the survey at any time. There is a risk of loss of confidentiality if your identity or answers are accidentally shared outside of the research team. We will do everything we can to prevent this from happening by using data security best practices, including storing data only on secured networks and keeping your identity separate from your survey answers, but we cannot 100% guarantee that there could be no loss of confidentiality. Will my information be kept private?The data for this study will be kept confidential to the extent allowed by federal and state law. Your responses will be returned to your local clinic and kept indefinitely by the research team. Under certain circumstances, information that identifies you may be released for internal and external reviews of this project. The results of this study may be published or presented at professional meetings, but the identities of all research participants will remain anonymous. Are there any costs or payments for being in this study?There is no cost to you for taking part in this study. You will receive a $100 gift card for completing the survey. You will be asked to provide your name and contact information to receive this payment.Who can I talk to if I have questions?If you have questions about this study or the information in this form, please contact the researcher, Dedra Buchwald, MD, at (206) 883-4740 or email dedra.buchwald@wsu.edu, or regular mail at: WSU/ IREACH 1100 Olive Way, Ste 1200 Seattle, WA 98101. If you have questions about your rights as a research participant, or would like to report a concern or complaint about this study, please contact the Washington State University Institutional Review Board at (509) 335-7646, or e-mail irb@wsu.edu, or regular mail at: Neill 427, PO Box 643143, Pullman, WA 99164-3143.What are my rights as a research study volunteer?Your participation in this research study is completely voluntary. There will be no penalty to you if you choose not to take part. You may choose not to answer specific questions or to stop participating at any time. You will be given a copy of this form for your records.By completing this survey, it means that you are voluntarily consenting to part of this research study and that you understand the potential benefits and risks that are involved.	descriptive

6	noteligible<br>Show the field ONLY if:<br>[age] < 18 or [aian] = '0'	Unfortunately, you are not eligible to participate in this study. Thank you for your time!	descriptive
7	concerts_study_info_and_consent_complete	Section Header: *Form Status*<br>Complete?	dropdown<br>0 Incomplete<br>1 Unverified<br>2 Complete

Instrument: **General Information** (general_information) Collapse

8	tribe	What is your primary tribal affiliation?	text
9	hispanic	Are you of Hispanic or Latino(a) origin?	yesno<br>1 Yes<br>0 No
10	sex_birth	What was your biological sex assigned at birth?	radio<br>1 Male<br>2 Female<br>3 Intersex<br>4 None of these describe me<br>5 Prefer not to answer
11	gender_identity	What term best expresses how you describe your gender identity?	radio<br>2 Woman<br>1 Man<br>3 Non-binary<br>4 Transgender man/Female-to-male (FTM)<br>5 Transgender woman/Male-to-female (MTF)<br>6 Gender non-binary/Genderqueer/Gender nonconforming<br>7 Agender<br>8 Bigender<br>9 None of these describe me
12	shazam_height	Section Header: *How tall are you without shoes?*	descriptive
13	height_feet	feet<br>*feet*	text (integer, Min: 0, Max: 10)
14	height_inches	inches<br>*inches*	text (integer, Min: 0, Max: 11)
15	weight	How much do you weigh without clothes or shoes?<br>*pounds*	text (integer, Min: 50, Max: 800)
16	educ	What is the highest level of education you have completed?	radio<br>1 Less than high school, no diploma<br>2 High school graduate or GED<br>3 Some college<br>4 Associate degree, 2 year degree, occupational, technical, or vocational program<br>5 College degree or higher

17	work	What is your current employment status?	radio<br>1 Working full time<br>2 Working part time<br>3 Temporarily laid off<br>4 Unemployed and looking for work<br>5 Unemployed and not looking for work<br>6 Retired<br>7 Disabled, either permanently or temporarily<br>8 Homemaker or stay at home parent<br>9 Student<br>10 Other
18	occup	What is or was your occupation?	text
19	employed_ew	Are you considered an essential worker? An essential worker is someone who was required to go to work even when stay at home orders were in place.	radio<br>1 Yes<br>0 No<br>888 Not currently working
20	marital	Are you currently...	radio<br>1 Married<br>2 Divorced<br>3 Widowed<br>4 Separated<br>5 Never married<br>6 A member of an unmarried couple
21	relation	Are you currently in a relationship?	yesno<br>1 Yes<br>0 No
22	household_famgen	What best describes your family at home?	radio<br>1 Just me<br>2 Living with spouse, no kids<br>3 Family including kids<br>4 Family with 3 generations (parents, children, grandchildren)<br>5 Family with 4 generations<br>888 None of these
23	housing	Are you currently living in transitional housing, a residential facility, staying in a shelter, or experiencing homelessness?	yesno<br>1 Yes<br>0 No
24	general_information_complete	Section Header: *Form Status*<br>Complete?	dropdown<br>0 Incomplete<br>1 Unverified<br>2 Complete

Instrument: **About Your Health** (about_your_health) Collapse

25	genhlth	Would you say your health in general is...	radio<br>1 Excellent<br>2 Very good<br>3 Good<br>4 Fair<br>5 Poor

26	brfss_obesity	Section Header: *Have you ever been told by a doctor, nurse, or other health care professional that you have any of the following health conditions? Please answer to the best of your knowledge. Please check one answer for each line.*<br>Obesity	radio (Matrix)<br>0 No<br>1 Yes
27	brfss_dm	Diabetes	radio (Matrix)<br>0 No<br>1 Yes
28	brfss_heart	Heart disease, for example congestive heart failure or heart attack	radio (Matrix)<br>0 No<br>1 Yes
29	brfss_hbp	High blood pressure	radio (Matrix)<br>0 No<br>1 Yes
30	brfss_asthma	Asthma or COPD	radio (Matrix)<br>0 No<br>1 Yes
31	brfss_liver	Liver disease, for example hepatitis or cirrhosis	radio (Matrix)<br>0 No<br>1 Yes
32	brfss_cancer	Cancer within the last 5 years (other than skin cancer)	radio (Matrix)<br>0 No<br>1 Yes
33	brfss_renal	Kidney disease	radio (Matrix)<br>0 No<br>1 Yes
34	brfss_strk	Stroke	radio (Matrix)<br>0 No<br>1 Yes
35	disability_deaf	Are you deaf, or do you have difficulty hearing?	yesno<br>1 Yes<br>0 No
36	disability_blind	Are you blind, or do you have serious difficulty seeing, even when you are wearing glasses?	yesno<br>1 Yes<br>0 No
37	disability_decisions	Because of a physical, mental, or emotional condition, do you have serious difficulty concentrating, remembering, or making decisions?	yesno<br>1 Yes<br>0 No
38	disability_walking	Do you have serious difficulty walking or climbing stairs?	yesno<br>1 Yes<br>0 No
39	disability_dress	Do you have difficulty dressing or bathing?	yesno<br>1 Yes<br>0 No
40	disability_errands	Because of a physical, mental, or emotional condition, do you have serious difficulty doing errands alone such as visiting a doctor's office or shopping?	yesno<br>1 Yes<br>0 No

41	insur	Do you have any of the following kinds of health insurance or coverage? Check all that apply.	checkbox<br>1 insur___1 Indian Health Service (IHS) or tribally run programs<br>2 insur___2 Insurance purchased directly by you or another family member<br>3 insur___3 Insurance through a current or former employer held by you or another family member<br>4 insur___4 Medicare<br>5 insur___5 Medicaid<br>6 insur___6 TRICARE or other military health insurance<br>7 insur___7 Veterans Affairs (VA)<br>8 insur___8 Other<br>9 insur___9 I don't have any health insurance
42	about_your_health_complete	Section Header: *Form Status*<br>Complete?	dropdown<br>0 Incomplete<br>1 Unverified<br>2 Complete
Instrument: **Community, Culture and Spirituality** (community_culture_and_spirituality)			Collapse
43	conn_comm_desc	We would like to know how connected you feel to your community, however you define it. Select the picture that best describes your relationship with your community. The less the circles overlap, the less connected you feel to your community; the more the circles overlap, the more connected you feel to your community.	descriptive
44	conn_commc		radio<br>1 1<br>2 2<br>3 3<br>4 4<br>5 5<br>6 6<br>Custom alignment: LV
45	lang	What language do you most often speak at home?	radio<br>1 American Indian or Alaska Native language<br>2 English<br>3 Spanish<br>4 Other
46	wol_aian	Section Header: *How much do you follow a ... Please check one answer for each line.*<br>American Indian or Alaska Native way of life?	radio (Matrix)<br>1 Not at All<br>2 A Little<br>3 Somewhat<br>4 A Lot
47	wol_white	White way of life?	radio (Matrix)<br>1 Not at All<br>2 A Little<br>3 Somewhat<br>4 A Lot

48	values_aian	Section Header: *How important is it that you keep... Please check one answer for each line.*<br>Your American Indian or Alaska Native identity, and your American Indian or Alaska Native values and practices?	radio (Matrix)<br>1 Not at All<br>2 A Little<br>3 Somewhat<br>4 A Lot
49	values_white	A White identity and White values and practices?	radio (Matrix)<br>1 Not at All<br>2 A Little<br>3 Somewhat<br>4 A Lot
50	spirit	Section Header: *Many people find that spirituality or some type of religion is important in their lives.*<br>How important is spirituality or some type of religion in your life?	radio<br>1 Not at all important<br>2 Somewhat important<br>3 Very important
51	spirit_time	How often do you spend time on such spiritual or religious practices?	radio<br>1 Very rarely or not at all<br>2 From time to time<br>3 Several times a month<br>4 Every day or almost every day
52	community_culture_and_spirituality_complete	Section Header: *Form Status*<br>Complete?	dropdown<br>0 Incomplete<br>1 Unverified<br>2 Complete
Instrument: **Lifestyle** (lifestyle)			Collapse
53	smoker_yn	Section Header: *These questions ask about your use of commercial tobacco, alcohol, and drugs to better understand how they may affect your health.Tobacco Use*<br>Have you smoked at least 100 cigarettes in your entire life? There are 20 cigarettes in a pack.	yesno<br>1 Yes<br>0 No
54	smoke_adultcurrent<br>Show the field ONLY if:<br>[smoker_yn] = '1'	Do you currently smoke?	radio<br>1 Every day<br>2 Some days<br>3 Not at all
55	exposure_smokemany<br>Show the field ONLY if:<br>[smoker_yn] = '1'	In the past month, how much did you smoke?	radio<br>1 A pack or more per day (a pack is 20 cigarettes)<br>2 Daily 10-19 cigarettes<br>3 Daily 4-9 cigarettes<br>4 Daily 1-3 cigarettes<br>5 Less than 1 cigarette per day
56	smoke_years<br>Show the field ONLY if:<br>[smoker_yn] = '1'	How many years have you smoked?<br>*years*	text (integer, Min: 0, Max: 99)
57	vaper_yn	Have you vaped at least 5 times in your life?	yesno<br>1 Yes<br>0 No
58	vape_adultcurrent<br>Show the field ONLY if:<br>[vaper_yn] = '1'	Do you currently vape?	radio<br>1 Every day<br>2 Some days<br>3 Not at all

59	exposure_vape<br>Show the field ONLY if:<br>[vaper_yn] = '1'	In the past month, how much did you vape?	radio<br>1 More than once a day<br>2 Daily or almost every day<br>3 3-4 days a week<br>4 1-3 days a week<br>5 Less than that
60	vaper_years<br>Show the field ONLY if:<br>[vaper_yn] = '1'	How many years have you vaped?<br>*years*	text (integer, Min: 0, Max: 99)
61	audit_freq	Section Header: *Alcohol Use*<br>How often did you have a drink containing alcohol in the past year?	radio<br>1 Never<br>2 Monthly or less<br>3 2 to 4 times a month<br>4 2 to 3 times a week<br>5 4 or more times a week
62	audit_daynum<br>Show the field ONLY if:<br>[audit_freq] = '2' or [audit_freq] = '3' or [audit_freq] = '4' or [audit_freq] = '5'	How many drinks did you have on a typical day when you were drinking in the past year?	radio<br>0 1 or 2 drinks<br>1 3 or 4 drinks<br>2 5 or 6 drinks<br>3 7 to 9 drinks<br>4 10 or more drinks
63	audit_6more<br>Show the field ONLY if:<br>[audit_freq] = '2' or [audit_freq] = '3' or [audit_freq] = '4' or [audit_freq] = '5'	How often do you have 6 or more drinks on one occasion?	radio<br>0 Never<br>1 Less than monthly<br>2 Monthly<br>3 Weekly<br>4 Daily or almost daily
64	sub_cannabis	Section Header: *Substance Use In the past 12 months, how often have you used the following? Please check one answer for each line. Next, indicate if use of each of the substances below caused any impairment. We define impairment as failing to do what was normally expected of you because of your use of that substance.*<br>Cannabis (marijuana, weed, hash, etc.) for reasons other than prescribed	radio<br>0 Never<br>1 A few times ever<br>2 Few times a year<br>3 Few times a month<br>4 Few times a week<br>5 Daily
65	sub_cannabis_impair<br>Show the field ONLY if:<br>[sub_cannabis] = '1' or [sub_cannabis] = '2' or [sub_cannabis] = '3' or [sub_cannabis] = '4' or [sub_cannabis] = '5'	Caused impairment?	radio<br>1 Yes<br>0 No
66	sub_rx	Prescription medications like stimulants (Ritalin, Concerta, Dexedrine, Adderall, diet pills, etc.)or opioids (fentanyl, oxycodone, OxyContin, Percocet, hydrocodone, Vicodin, methadone, buprenorphine, etc.)for reasons other than prescribed	radio<br>0 Never<br>1 A few times ever<br>2 Few times a year<br>3 Few times a month<br>4 Few times a week<br>5 Daily

67	sub_rx_impair<br>Show the field ONLY if:<br>[sub_rx] = '1' or [sub_rx] = '2' or [sub_rx] = '3' or [sub_rx] = '4' or [sub_rx] = '5'	Caused impairment?	radio<br>1 Yes<br>0 No
68	sub_illicit	Illicit drugs (cocaine, methamphetamine, inhalants, sedatives, hallucinogens, heroin, opium, etc.)	radio<br>0 Never<br>1 A few times ever<br>2 Few times a year<br>3 Few times a month<br>4 Few times a week<br>5 Daily
69	sub_illicit_impair<br>Show the field ONLY if:<br>[sub_illicit] = '1' or [sub_illicit] = '2' or [sub_illicit] = '3' or [sub_illicit] = '4' or [sub_illicit] = '5'	Caused impairment?	radio<br>1 Yes<br>0 No
70	lifestyle_complete	Section Header: *Form Status*<br>Complete?	dropdown<br>0 Incomplete<br>1 Unverified<br>2 Complete
Instrument: **Individual And Structural Determinants Of Health** (individual_and_structural_determinants_of_health) Collapse			
71	income_desc	The ability to manage the stress and hardship of the COVID-19 pandemic is related in part to the resources available to a person and his or her family. Please answer the following questions to the best of your knowledge.	descriptive
72	income	In the past 12 months, what was your total household income including wages, Social Security, disability, and child support?	radio<br>1 Under $10,000<br>2 Between $10,000 and $14,999<br>3 Between $15,000 and $19,999<br>4 Between $20,000 and $24,999<br>5 Between $25,000 and $29,999<br>6 Between $30,000 and $34,999<br>7 Between $35,000 and $39,999<br>8 Between $40,000 and $44,999<br>9 Between $45,000 and $49,999<br>10 Between $50,000 and $59,999<br>11 Between $60,000 and $74,999<br>12 Between $75,000 and $99,999<br>13 Between $100,000 and $124,999<br>14 Between $125,000 and $149,999<br>15 More than $150,000
73	covid_payment	Since the beginning of the COVID-19 pandemic in February 2020, have you received any payments from your tribe specifically for COVID-19 relief?	yesno<br>1 Yes<br>0 No
74	reg_payment	Do you receive any regular form of payment from your tribe, for example, dividends from a Native corporation or per capita cash transfer?	yesno<br>1 Yes<br>0 No

75	payment_amt<br>Show the field ONLY if:<br>[reg_payment] = '1'	To the best of your knowledge, what is the total amount of money you received from your tribe or Native corporation in the past year?<br>*$*	text
76	payment_freq<br>Show the field ONLY if:<br>[reg_payment] = '1'	How often do you receive payments?	radio<br>1 Once a year<br>2 Several times a year<br>3 Monthly
77	covpay_supp<br>Show the field ONLY if:<br>[reg_payment] = '1'	Since the beginning of the COVID-19 pandemic in February 2020, have you spent any of the money you received from your tribe or Native corporation on items like face masks, cleaning supplies, or hand sanitizer to keep you or someone else protected from COVID-19?	yesno<br>1 Yes<br>0 No
78	covpay_suppamt<br>Show the field ONLY if:<br>[reg_payment] = '1' and [covpay_supp]='1'	In total, how much of the money you received from your tribe or Native corporation did you spend on these items?	radio<br>1 0 to $24.99<br>2 $25 to $49.99<br>3 $50 to $99.99<br>4 $100 or more
79	primarycare	Section Header: *Access to Health Care*<br>Do you have a primary care provider or doctor, nurse, or other health care professional you see regularly?	yesno<br>1 Yes<br>0 No
80	televisit	Have you ever had a visit with a doctor, nurse, or other health care professional using your computer, smart phone, or tablet?	radio<br>1 Yes<br>0 No<br>888 I don't have a computer, smart phone, or tablet
81	internet	Do you have regular Internet access using... Check all that apply	checkbox<br>1 internet___1 Your own computer<br>2 internet___2 Someone else's computer or a computer outside of your home<br>3 internet___3 Your smart phone or tablet<br>4 internet___4 I don't have regular Internet access
82	text_phone	Can you receive text messages on your phone?	yesno<br>1 Yes<br>0 No
83	hcplace	Where do you usually go when you need health care?	radio<br>1 Doctor's office or health center<br>2 Walk-in clinic, urgent care center, or retail clinic in a pharmacy or grocery store<br>3 Emergency room<br>4 VA Medical Center or VA outpatient clinic<br>5 Some other place<br>6 Many different places<br>7 I don't go anywhere
84	traveltime	About how long does it take for you to travel to Hunter Health Clinic from your home?<br>*minutes*	text (integer)

85	travelmode	What mode of transportation do you usually use to get to Hunter Health Clinic?	radio<br>1 Car - I drive myself<br>2 Car - I get a ride with someone I know<br>3 Bus<br>4 Taxi or ride share<br>5 Bike<br>6 Walk
86	hosp	In the past 12 months, have you been hospitalized overnight?	yesno<br>1 Yes<br>0 No
87	meddelay_cost	In the past 12 months, have you delayed getting medical care because of the cost?	yesno<br>1 Yes<br>0 No
88	nomed_cost	In the past 12 months, was there any time when you needed medical care, but did not get it because of the cost?	yesno<br>1 Yes<br>0 No
89	hlit_read	Section Header: *Health Literacy*<br>How often do you have someone help you read clinic or health related materials?	radio<br>5 Always<br>4 Often<br>3 Sometimes<br>2 Rarely<br>1 Never
90	hlit_forms	How confident are you filling out medical forms by yourself?	radio<br>1 Not at all<br>2 A little bit<br>3 Somewhat<br>4 Quite a bit<br>5 Extremely
91	hlit_wrttn	How often do you have difficulty learning about your health because of difficulty understanding written information?	radio<br>5 Always<br>4 Often<br>3 Sometimes<br>2 Rarely<br>1 Never
92	hlit_told	How often do you have a problem understanding what is told to you about a health condition?	radio<br>5 Always<br>4 Often<br>3 Sometimes<br>2 Rarely<br>1 Never
93	individual_and_structural_determinants_of_health_complete	Section Header: *Form Status*<br>Complete?	dropdown<br>0 Incomplete<br>1 Unverified<br>2 Complete

Instrument: **Food Insecurity** (food_insecurity) Collapse

94	foodins_notenough	Section Header: *In the past 12 months, please indicate how often each statement has been true for you and the other members of your household.*<br>In the past 12 months, the food that my household bought just didn't last, and there wasn't enough money to get more.	radio<br>2 Often true<br>1 Sometimes true<br>0 Never true
95	foodins_afford	In the past 12 months, my household couldn't afford to eat balanced (healthy) meals.	radio<br>2 Often true<br>1 Sometimes true<br>0 Never true
96	foodins_skip	In the past 12 months, did you or anyone in your household ever cut down the size of your meals or skip meals because there wasn't enough money for food?	yesno<br>1 Yes<br>0 No
97	foodins_skip_freq<br>Show the field ONLY if:<br>[foodins_skip] = '1'	In the past 12 months, how often did this happen?	radio<br>2 Almost every month<br>1 Some months but not every month<br>0 Only 1 or 2 months
98	foodins_eatless	In the past 12 months, did you ever eat less than you felt you should because there wasn't enough money to buy food?	yesno<br>1 Yes<br>0 No
99	foodins_noteat	In the past 12 months, were you ever hungry but didn't eat because you couldn't afford enough food?	yesno<br>1 Yes<br>0 No
100	jobsec_laidoff	Section Header: *Job Security*<br>In the next 12 months, how likely do you think it is that you will lose your job or be laid off?	radio<br>1 Very likely<br>2 Somewhat likely<br>3 Not at all likely<br>888 Not currently working
101	jobsec_newjob	How easy would it be for you to find a job with another employer with about the same income and benefits you now have?	radio<br>1 Very likely<br>2 Somewhat likely<br>3 Not at all likely<br>4 Very easy<br>5 Somewhat easy<br>6 Not at all easy<br>888 Not currently working<br>Field Annotation: @HIDECHOICE="1,2,3"
102	food_insecurity_complete	Section Header: *Form Status*<br>Complete?	dropdown<br>0 Incomplete<br>1 Unverified<br>2 Complete
Instrument: **Discrimination, Distress & Trauma** (discrimination_distress_trauma)			Collapse
103	pds_school	Section Header: *Discrimination Think about your identity as an American Indian or Alaska Native person. How often have any of the following things below happened to you because of being American Indian or Alaska Native? Please check one answer for each line.*<br>Have you been treated unfairly by teachers or other staff at school?	radio (Matrix)<br>1 Never<br>2 Rarely<br>3 Sometimes<br>4 Often<br>5 Very often

104	pds_job	Have others thought you couldn't do things or handle a job?	radio (Matrix)<br>1 Never<br>2 Rarely<br>3 Sometimes<br>4 Often<br>5 Very often
105	pds_threat	Have others threatened to hurt you?	radio (Matrix)<br>1 Never<br>2 Rarely<br>3 Sometimes<br>4 Often<br>5 Very often
106	pds_hurt	Have others actually hurt you or tried to hurt you?	radio (Matrix)<br>1 Never<br>2 Rarely<br>3 Sometimes<br>4 Often<br>5 Very often
107	pds_police	Have policemen or security officers been unfair to you?	radio (Matrix)<br>1 Never<br>2 Rarely<br>3 Sometimes<br>4 Often<br>5 Very often
108	pds_threatprop	Have others threatened to damage your property?	radio (Matrix)<br>1 Never<br>2 Rarely<br>3 Sometimes<br>4 Often<br>5 Very often
109	pds_propdam	Have others actually damaged your property?	radio (Matrix)<br>1 Never<br>2 Rarely<br>3 Sometimes<br>4 Often<br>5 Very often
110	pds_outsider	Have others made you feel like an outsider who doesn't fit in because of your dress, speech, or other characteristics related to your ethnicity?	radio (Matrix)<br>1 Never<br>2 Rarely<br>3 Sometimes<br>4 Often<br>5 Very often
111	pds_cowork	Have you been treated unfairly by co-workers or classmates?	radio (Matrix)<br>1 Never<br>2 Rarely<br>3 Sometimes<br>4 Often<br>5 Very often

112	pds_dishon	Have others hinted you are dishonest or can't be trusted?	radio (Matrix)<br>1 Never<br>2 Rarely<br>3 Sometimes<br>4 Often<br>5 Very often
113	pds_back	Have people been nice to your face, but said bad things behind your back?	radio (Matrix)<br>1 Never<br>2 Rarely<br>3 Sometimes<br>4 Often<br>5 Very often
114	pds_difflang	Have people who speak a different language made you feel like an outsider?	radio (Matrix)<br>1 Never<br>2 Rarely<br>3 Sometimes<br>4 Often<br>5 Very often
115	pds_clean	Have others hinted you must not be clean?	radio (Matrix)<br>1 Never<br>2 Rarely<br>3 Sometimes<br>4 Often<br>5 Very often
116	pds_distrust	Have people not trusted you?	radio (Matrix)<br>1 Never<br>2 Rarely<br>3 Sometimes<br>4 Often<br>5 Very often
117	pds_lazy	Has it been said that you must be lazy?	radio (Matrix)<br>1 Never<br>2 Rarely<br>3 Sometimes<br>4 Often<br>5 Very often
118	pds_ignore	Have others ignored you or not paid attention to you?	radio (Matrix)<br>1 Never<br>2 Rarely<br>3 Sometimes<br>4 Often<br>5 Very often
119	pds_boss	Has your boss or supervisor been unfair to you?	radio (Matrix)<br>1 Never<br>2 Rarely<br>3 Sometimes<br>4 Often<br>5 Very often

120	kpds_nrvs	Section Header: *Distress The next questions ask about how you have been feeling in the past month. Please check one answer for each question.*<br>In the past month, about how often did you feel nervous?	radio<br>5 All of the time<br>4 Most of the time<br>3 Some of the time<br>2 A little of the time<br>1 None of the time
121	kpds_hplss	In the past month, about how often did you feel hopeless?	radio<br>5 All of the time<br>4 Most of the time<br>3 Some of the time<br>2 A little of the time<br>1 None of the time
122	kpds_rstlss	In the past month, about how often did you feel restless or fidgety?	radio<br>5 All of the time<br>4 Most of the time<br>3 Some of the time<br>2 A little of the time<br>1 None of the time
123	kpds_nocheer	In the past month, about how often did you feel so depressed that nothing could cheer you up?	radio<br>5 All of the time<br>4 Most of the time<br>3 Some of the time<br>2 A little of the time<br>1 None of the time
124	kpds_effort	In the past month, about how often did you feel that everything was an effort?	radio<br>5 All of the time<br>4 Most of the time<br>3 Some of the time<br>2 A little of the time<br>1 None of the time
125	kpds_wrthlss	In the past month, about how often did you feel worthless?	radio<br>5 All of the time<br>4 Most of the time<br>3 Some of the time<br>2 A little of the time<br>1 None of the time
126	ptsd_dreams	Section Header: *Trauma Now we'd like to ask you questions about some thoughts or feelings you may have had. You might be wondering "What is trauma?" By trauma we mean a frightening or upsetting event like:• A serious accident• A natural disaster like an earthquake or fire• Loss of a loved one• Violence (seeing someone get hurt badly or getting hurt badly yourself)• Abuse (seeing someone abusing someone that you care about or being abused yourself)• Being in combat or a war zone Have you ever had a trauma like the examples above that was so frightening, horrible, or upsetting that you...*<br>Have had dreams about it or thought about it when you did not want to?	yesno<br>1 Yes<br>0 No
127	ptsd_avoid	Tried hard not to think about it or went out of your way to avoid situations that reminded you of it?	yesno<br>1 Yes<br>0 No
128	ptsd_onguard	Were constantly on guard, watchful, or easily startled?	yesno<br>1 Yes<br>0 No

129	ptsd_numb	Felt numb or detached from others, activities, or your surroundings?	yesno<br>1 Yes<br>0 No
130	discrimination_distress_trauma_complete	Section Header: *Form Status*<br>Complete?	dropdown<br>0 Incomplete<br>1 Unverified<br>2 Complete
Instrument: **Stress & Resilience** (stress_resilience)			Collapse
131	pss_nocontrol	Section Header: *Stress The questions in this scale ask you about your feelings and thoughts in the past month. In each case, please indicate your response by selecting the answer that represents how often you felt or thought a certain way. Please check one answer for each line.*<br>In the past month, how often have you felt that you were unable to control the important things in your life?	radio (Matrix)<br>0 Never<br>1 Almost Never<br>2 Sometimes<br>3 Fairly Often<br>4 Very Often
132	pss_prob	In the past month, how often have you felt confident about your ability to handle your personal problems?	radio (Matrix)<br>0 Never<br>1 Almost Never<br>2 Sometimes<br>3 Fairly Often<br>4 Very Often
133	pss_way	In the past month, how often have you felt that things were going your way?	radio (Matrix)<br>0 Never<br>1 Almost Never<br>2 Sometimes<br>3 Fairly Often<br>4 Very Often
134	pss_notovercome	In the past month, how often have you felt difficulties were piling up so high that you could not overcome them?	radio (Matrix)<br>0 Never<br>1 Almost Never<br>2 Sometimes<br>3 Fairly Often<br>4 Very Often
135	stress_now	How stressed are you feeling right now?	radio<br>1 1 Not stressed at All<br>2 2<br>3 3<br>4 4<br>5 5 Very Stressed<br>Custom alignment: LH
136	brs_bounce	Section Header: *Resilience Please indicate the extent to which you agree with each of the following statements by using the following scale: strongly disagree, disagree, neutral, agree, strongly agree. Please check one answer for each line.*<br>I tend to bounce back quickly after hard times	radio (Matrix)<br>1 Strongly disagree<br>2 Disagree<br>3 Neutral<br>4 Agree<br>5 Strongly agree

137	brs_hardtime	I have a hard time making it through stressful events	radio (Matrix)<br>1 Strongly disagree<br>2 Disagree<br>3 Neutral<br>4 Agree<br>5 Strongly agree
138	brs_notlong	It does not take me long to recover from a stressful event	radio (Matrix)<br>1 Strongly disagree<br>2 Disagree<br>3 Neutral<br>4 Agree<br>5 Strongly agree
139	brs_snapback	It is hard for me to snap back when something bad happens	radio (Matrix)<br>1 Strongly disagree<br>2 Disagree<br>3 Neutral<br>4 Agree<br>5 Strongly agree
140	brs_through	I usually come through difficult times with little trouble	radio (Matrix)<br>1 Strongly disagree<br>2 Disagree<br>3 Neutral<br>4 Agree<br>5 Strongly agree
141	brs_longtime	I tend to take a long time to get over setbacks in my life	radio (Matrix)<br>1 Strongly disagree<br>2 Disagree<br>3 Neutral<br>4 Agree<br>5 Strongly agree
142	stress_resilience_complete	Section Header: *Form Status*<br>Complete?	dropdown<br>0 Incomplete<br>1 Unverified<br>2 Complete
Instrument: **COVID-19 Your Knowledge And Perceptions** (covid19_your_knowledge_and_perceptions) Collapse			
143	covidknow_desc	To the best of your knowledge, please answer the following questions about COVID-19.	descriptive
144	covidkp_age	What age group is most likely to die from COVID-19?	radio<br>1 Children<br>2 Young adults<br>3 Middle-age adults<br>4 Older adults
145	covidkp_chronic	Are people with chronic health problems more likely to die from COVID-19 than people in good health?	yesno<br>1 Yes<br>0 No
146	covidkp_dthrate	What percentage of people who get COVID-19 end up dying from it?<br>*%*	text (number, Min: 0, Max: 100)

147	covidkp_prtct_mask	Section Header: *Which of the following actions can protect you against getting infected with COVID-19? Please check 'True' or 'False' for each line.*<br>Wearing a face mask	radio (Matrix)<br>1 True<br>0 False
148	covidkp_prtct_vac	Getting a vaccination against influenza (the flu)	radio (Matrix)<br>1 True<br>0 False
149	covidkp_prtct_mthw	Gargling mouthwash	radio (Matrix)<br>1 True<br>0 False
150	covidkp_prtct_wsh	Washing your hands	radio (Matrix)<br>1 True<br>0 False
151	covidkp_prtct_avdcc	Avoid close contact with people who are sick	radio (Matrix)<br>1 True<br>0 False
152	covidkp_prtct_socd	Social distancing or standing apart from other people	radio (Matrix)<br>1 True<br>0 False
153	covidkp_prtct_antib	Taking antibiotics	radio (Matrix)<br>1 True<br>0 False
154	covidkp_prtct_hddry	Using a hand dryer	radio (Matrix)<br>1 True<br>0 False
155	covidkp_prtct_avdface	Avoiding touching your eyes, nose, and mouth with unwashed hands	radio (Matrix)<br>1 True<br>0 False
156	covidkp_infct_fec	Section Header: *Are each of the following a main way that people are currently getting infected with COVID-19? Please check only one answer for each line.*<br>Fecal contaminants in drinking water	radio (Matrix)<br>1 Yes<br>0 No
157	covidkp_infct_food	Unhygienic preparation of food	radio (Matrix)<br>1 Yes<br>0 No
158	covidkp_infct_sex	Sexual intercourse or sharing of needles for drug use	radio (Matrix)<br>1 Yes<br>0 No
159	covidkp_infct_mosq	Mosquito bites	radio (Matrix)<br>1 Yes<br>0 No
160	covidkp_infct_sal	Droplets of saliva from infected people	radio (Matrix)<br>1 Yes<br>0 No
161	covidkp_infct_bfl	Directly coming into touch with someone's bodily fluids like blood, vomit, or sweat	radio (Matrix)<br>1 Yes<br>0 No
162	covidkp_dist	About how far away from another person do you think you should stand to protect yourself from getting infected with COVID-19?<br>*feet*	text (integer)

163	covidkp_symp_nsbld	Section Header: *What are common signs or symptoms of a COVID-19 infection? Please check 'True' or 'False' for each line.*<br>Nose bleeds	radio (Matrix)<br>1 True<br>0 False
164	covidkp_symp_cgh	Cough	radio (Matrix)<br>1 True<br>0 False
165	covidkp_symp_fvr	Fever or chills	radio (Matrix)<br>1 True<br>0 False
166	covidkp_symp_pain	Muscle pain, headache or sore throat	radio (Matrix)<br>1 True<br>0 False
167	covidkp_symp_tste	Loss of taste and smell	radio (Matrix)<br>1 True<br>0 False
168	covidkp_symp_rash	Skin rash	radio (Matrix)<br>1 True<br>0 False
169	covidkp_symp_const	Constipation	radio (Matrix)<br>1 True<br>0 False
170	covidkp_symp_brth	Shortness of breath	radio (Matrix)<br>1 True<br>0 False
171	covidkp_symp_urin	Frequent urination	radio (Matrix)<br>1 True<br>0 False
172	covid_ser	COVID-19 is a serious disease.	yesno<br>1 Yes<br>0 No
173	covid_risk	I am at risk of being infected with COVID-19.	yesno<br>1 Yes<br>0 No
174	covid_spread	COVID-19 is spreading in the community where I live.	yesno<br>1 Yes<br>0 No
175	covid19_your_knowledge_and_perceptions_complete	Section Header: *Form Status*<br>Complete?	dropdown<br>0 Incomplete<br>1 Unverified<br>2 Complete

Instrument: **COVID-19 And You** (covid19_and_you) Collapse

176	covid_postpone	Section Header: *Now we want to ask you some questions about COVID-19, the pandemic, and how it has affected your life.*<br>Since the beginning of the COVID-19 pandemic in February 2020, have you needed to postpone any medical procedures?	yesno<br>1 Yes<br>0 No
177	covid_notest	Have you had an illness that you believe was COVID-19 but did not get tested?	yesno<br>1 Yes<br>0 No

178	covid_notest_why<br>Show the field ONLY if:<br>[covid_notest] = '1'	Why did you not get tested? Check all that apply.	checkbox<br>1 covid_notest_why___1 I did not know where to get tested<br>2 covid_notest_why___2 The kind of test or the place where I could get tested was not culturally or socially acceptable to me<br>3 covid_notest_why___3 I could not get to a place that offered testing at all or in a timely fashion<br>4 covid_notest_why___4 I could not pay for testing or for the help I needed to get tested<br>5 covid_notest_why___5 I didn't think the benefit of getting tested was worth it
179	covid_drpos	Has a doctor, nurse or other health care professional ever told you that you were infected with or have COVID-19?	yesno<br>1 Yes<br>0 No
180	covid_dreasy<br>Show the field ONLY if:<br>[covid_drpos] = '1'	How easy or difficult was it for you to see or talk to a doctor, nurse, or other health care professional when you were infected or sick with COVID-19?	radio<br>1 Very difficult<br>2 Somewhat difficult<br>3 Somewhat easy<br>4 Very easy
181	covid_nomedcare<br>Show the field ONLY if:<br>[covid_drpos] = '1'	When you were infected or sick with COVID-19, was there a time that you needed medical care from a doctor, nurse, or other health care professional and couldn't get it?	yesno<br>1 Yes<br>0 No
182	covid_stayhome<br>Show the field ONLY if:<br>[covid_drpos] = '1'	When you were infected or sick with COVID-19, did you stay home from work, school, and activities outside your home for even one day?	yesno<br>1 Yes<br>0 No
183	covid_hometime<br>Show the field ONLY if:<br>[covid_drpos] = '1' AND [covid _stayhome] = '1'	How long did you stay home from work, school, or other activities outside your home?	radio<br>1 Until I felt well enough to participate in work, school, or other activities, but did not notice whether I had a fever at that time<br>2 Until just when my fever went away<br>3 For at least 24 hours after my fever went away<br>4 For 7 to 14 days to quarantine
184	covid_distancehh<br>Show the field ONLY if:<br>[covid_drpos] = '1'	While you were infected or sick with COVID-19, were you able to keep your distance from other people in your household?	yesno<br>1 Yes<br>0 No
185	covid_distancehh_unable<br>Show the field ONLY if:<br>[covid_drpos] = '1' and [covid_ distancehh] = '0'	While infected or sick with COVID-19, what was the main reason you were unable to keep your distance from other people in your household?	radio<br>1 I am the primary caretaker of my home<br>2 I needed help from other people in my home<br>3 The layout of my home made it hard<br>4 I needed to be around others in my home for my mental health<br>5 I did not feel the need to keep my distance<br>6 Other:
186	covid_distancehh_unableoth<br>Show the field ONLY if:<br>[covid_distancehh_unable] = '6'	Other:	text

187	covid_er<br>Show the field ONLY if:<br>[covid_drpos] = '1'	Did you go to an emergency room because you were infected or sick with COVID-19?	yesno<br>1 Yes<br>0 No
188	covid_hospadmit<br>Show the field ONLY if:<br>[covid_drpos] = '1'	Were you admitted to a hospital because of COVID-19?	yesno<br>1 Yes<br>0 No
189	covid_numtests	How many times have you been tested for COVID-19?	radio<br>0 Never<br>1 1 time<br>2 2 times<br>3 3 or more times
190	covid_testdiff<br>Show the field ONLY if:<br>[covid_numtests] = '1' or [covid_numtests] = '2' or [covid_numtests] = '3'	How easy or difficult was it for you to get tested for COVID-19?	radio<br>1 Very difficult<br>2 Somewhat difficult<br>3 Somewhat easy<br>4 Very easy
191	covid_testpos<br>Show the field ONLY if:<br>[covid_numtests] = '1' or [covid_numtests] = '2' or [covid_numtests] = '3'	Did any test indicate that you had COVID-19?	radio<br>1 Yes<br>0 No<br>2 I am waiting for the results
192	covid_testdate<br>Show the field ONLY if:<br>([covid_numtests] = '1' or [covid_numtests] = '2' or [covid_numtests] = '3') and [covid_testpos]='1'	What was the date of your first positive test?	text (date_mdy)<br>Field Annotation: @HIDEBUTTON
193	covid_resulttime<br>Show the field ONLY if:<br>([covid_numtests] = '1' or [covid_numtests] = '2' or [covid_numtests] = '3') and ([covid_testpos] = '1' or [covid_testpos] = '0')	About how long did it take to get your COVID-19 test results back?	radio<br>1 Less than 1 hour<br>2 1 hour to less than 12 hours<br>3 12 hours to less than 24 hours<br>4 1 to 2 days<br>5 3 to 4 days<br>6 More than 4 days
194	covid_testquar<br>Show the field ONLY if:<br>[covid_numtests] = '1' or [covid_numtests] = '2' or [covid_numtests] = '3'	Did you quarantine in between getting the COVID-19 test and receiving your results?	yesno<br>1 Yes<br>0 No
195	covid_hhdrpos	Not including yourself, has anyone in your household been told by a doctor, nurse or health care professional that they have COVID-19?	yesno<br>1 Yes<br>0 No
196	covid_hhtestpos<br>Show the field ONLY if:<br>[covid_hhdrpos] = '1'	Not including yourself, how many people in your household have had a positive test?	radio<br>1 1<br>2 2<br>3 3 or more
197	covid_sufftest	To the best of your knowledge, do you think there has been enough COVID-19 testing in your community for everyone who wants it, or has your community had a shortage of tests?	radio<br>1 Enough for anyone who wants a test<br>2 Had a shortage

198	covidresp_wash	Section Header: *Next is a list of things that some people are doing in response to the COVID-19 pandemic. Have you done any of the following in your daily routine at home or at work? Since the beginning of the COVID-19 pandemic in February 2020, have you done any of the following in response to the pandemic? Please check one answer for each line.*<br>Washed your hands more frequently	radio (Matrix)<br>1 Yes<br>0 No
199	covidresp_hsani	Used hand sanitizer more frequently	radio (Matrix)<br>1 Yes<br>0 No
200	covidresp_maskpp	Wore a mask in public places	radio (Matrix)<br>1 Yes<br>0 No
201	covidresp_tchface	Tried to keep from touching your eyes, nose, and mouth	radio (Matrix)<br>1 Yes<br>0 No
202	covidres_vitam	Taken vitamins or herbal supplements beyond the usual amount you take	radio (Matrix)<br>1 Yes<br>0 No
203	covidres_avdsym	Taken any steps to avoid being near someone who has a fever, cough, sore throat, or similar symptoms	radio (Matrix)<br>1 Yes<br>0 No
204	covidres_avdeld	Avoided visiting elderly family and friends	radio (Matrix)<br>1 Yes<br>0 No
205	covidres_sixft	Tried to stay 6 feet apart when socializing with people outside of your household	radio (Matrix)<br>1 Yes<br>0 No
206	covidres_cvrmth	More frequently covered your mouth and nose with a tissue when coughing or sneezing	radio (Matrix)<br>1 Yes<br>0 No
207	covidres_elbow	More frequently coughed or sneezed into your elbow or shoulder	radio (Matrix)<br>1 Yes<br>0 No
208	covidres_clean	More frequently cleaned or disinfected your home or workspace	radio (Matrix)<br>1 Yes<br>0 No
209	covidres_strng	Used additional or stronger cleaners or disinfectants than you normally use	radio (Matrix)<br>1 Yes<br>0 No
210	covidres_doc	Talked to a doctor, nurse, or other health care professional about what you could do to protect yourself or your family from getting infected or sick with COVID-19	radio (Matrix)<br>1 Yes<br>0 No
211	covidres_avdgthr	Avoided places where many people are gathered together, including movies, sporting events, concerts, shopping areas, indoor restaurants, or religious services	radio (Matrix)<br>1 Yes<br>0 No
212	covidres_limitpt	Section Header: *Since the beginning of the COVID-19 pandemic in February 2020, have you done any of the following in response to the pandemic? Please check one answer for each line.*<br>Limited your use of public transportation, buses, and trains	radio (Matrix)<br>1 Yes<br>0 No<br>888 Does not apply to me

213	covidres_avdtrv	Avoided air travel	radio (Matrix)<br>1 Yes<br>0 No<br>888 Does not apply to me
214	covidres_stayhm	Stayed home from work to avoid contact with co-workers who might be infected or sick with COVID-19 disease	radio (Matrix)<br>1 Yes<br>0 No<br>888 Does not apply to me
215	covidres_chldhm	Kept your children home from school even when they were not sick in order to avoid contact with children who might be infected or sick with COVID-19 disease	radio (Matrix)<br>1 Yes<br>0 No<br>888 Does not apply to me
216	covid19_and_you_complete	Section Header: *Form Status*<br>Complete?	dropdown<br>0 Incomplete<br>1 Unverified<br>2 Complete
	Instrument: **COVID-19 Impact On Your Life** (covid19_impact_on_your_life)		Collapse
217	covidlf_income	Since the beginning of the COVID-19 pandemic in February 2020, has your income	radio<br>3 Increased<br>2 Stayed about the same<br>1 Decreased
218	covidlf_res	Since the beginning of the COVID-19 pandemic in February 2020, how many times have you traveled to..... Your reservation, tribal homelands, or village?<br>*times*	text (integer)
219	covidlf_reslngd<br>Show the field ONLY if:<br>[covidlf_res] > 0	What was the longest time you stayed?<br>*days*	text (integer)
220	covidlf_resshtd<br>Show the field ONLY if:<br>[covidlf_res] > 0	What was the shortest time you stayed?<br>*days*	text (integer)
221	covidlf_othres	Since the beginning of the COVID-19 pandemic in February 2020, how many times have you traveled to... Other reservations, tribal homelands, or villages<br>*times*	text (integer)
222	covidlf_othreslngd<br>Show the field ONLY if:<br>[covidlf_othres] > 0	What was the longest time you stayed?<br>*days*	text (integer)
223	covidlf_othresshtd<br>Show the field ONLY if:<br>[covidlf_othres] > 0	What was the shortest time you stayed?<br>*days*	text (integer)
224	covidlf_trvl<br>Show the field ONLY if:<br>[covidlf_res] > 0	What is the main reason you traveled to your reservation, tribal homelands, or village?	radio<br>1 To get health care<br>2 To be close to family<br>3 To attend a ceremony, traditional foods gathering, funeral, or other cultural event<br>4 To take care of a family member or friend with COVID-19<br>5 To provide care for family or friends who were not sick with COVID-19 but were experiencing hardships because of the pandemic<br>6 Other

225	covidlf_ctelder	Are you providing additional caretaking of elders due to the COVID-19 pandemic, for example, delivering groceries or providing transportation?	yesno<br>1 Yes<br>0 No
226	covidlf_ctchld	Are you providing additional caretaking or home schooling of children due to the COVID-19 pandemic?	yesno<br>1 Yes<br>0 No
227	shazam_sleep	Section Header: *In the past month, how many hours of sleep do you get in a 24-hour period?*	descriptive
228	sleep_hrs	hours<br>*hours*	text (integer, Min: 0, Max: 24)
229	sleep_min	minutes<br>*minutes*	text (integer, Min: 0, Max: 59)
230	trouble_sleep	Over the past 2 weeks, how many days have you had trouble falling asleep or sleeping too much?<br>*days*	text (integer, Min: 0, Max: 14)
231	exercise_days	In the past month, about how many days per week did you exercise?	radio<br>1 No days<br>2 1 to 2 days<br>3 3 to 4 days<br>4 5 or more days
232	exercise_time	In the past month, how much time do you spend exercising on days that you exercised?	radio<br>1 None<br>2 1 to 30 minutes<br>3 More than 30 minutes up to 1 hour<br>4 More than 1 hour
233	exercise_comp	How does this compare with the amount of time you typically exercised before the COVID-19 pandemic?	radio<br>3 More time<br>2 Unchanged amount of time<br>1 Less time
234	tradmed_covid	Since the beginning of the COVID-19 pandemic in February 2020, has your use of traditional medicine or healing practices...	radio<br>3 Increased<br>2 Stayed about the same<br>1 Decreased<br>888 I don't use traditional medicine or healing practices
235	covid19_impact_on_your_life_complete	Section Header: *Form Status*<br>Complete?	dropdown<br>0 Incomplete<br>1 Unverified<br>2 Complete
Instrument: **COVID-19 Impact On Your Wellbeing** (covid19_impact_on_your_wellbeing)			Collapse
236	covidwb_infect	How concerned are you about getting infected or sick with COVID-19?	radio<br>5 Extremely concerned<br>4 Very concerned<br>3 Moderately concerned<br>2 A little concerned<br>1 Not at all concerned

237	covidwb_exptrans	How concerned are you about being exposed to COVID-19 while using your typical transportation?	radio<br>5 Extremely concerned<br>4 Very concerned<br>3 Moderately concerned<br>2 A little concerned<br>1 Not at all concerned<br>888 Not using transportation
238	covidwb_expwrk	How concerned are you about being exposed to COVID-19 at your workplace?	radio<br>5 Extremely concerned<br>4 Very concerned<br>3 Moderately concerned<br>2 A little concerned<br>1 Not at all concerned<br>888 Not currently working or retired
239	covidwb_wgs	Section Header: *Have you experienced any of the following difficulties due to the COVID-19 pandemic? Please answer Yes or No for each difficulty.*<br>Having wages or work hours reduced	radio (Matrix)<br>1 Yes<br>0 No
240	covidwb_losejob	Losing my job	radio (Matrix)<br>1 Yes<br>0 No
241	covidwb_chldcare	Finding childcare	radio (Matrix)<br>1 Yes<br>0 No
242	covidwb_food	Getting food	radio (Matrix)<br>1 Yes<br>0 No
243	covidwb_supp	Getting hand sanitizer, masks, or cleaning supplies	radio (Matrix)<br>1 Yes<br>0 No
244	covidwb_rx	Getting routine or essential medications	radio (Matrix)<br>1 Yes<br>0 No
245	covidwb_transp	Finding transportation	radio (Matrix)<br>1 Yes<br>0 No
246	covidwb_hcacc	Accessing health care	radio (Matrix)<br>1 Yes<br>0 No
247	covidwb_lose	Losing a loved one to COVID-19 disease	radio (Matrix)<br>1 Yes<br>0 No
248	covidwb_isol	Being isolated from others who are important to me	radio (Matrix)<br>1 Yes<br>0 No
249	covidwb_missevnt	Missing important events (for example, funeral, ceremony, spiritual or religious activities)	radio (Matrix)<br>1 Yes<br>0 No

250	covidwb_nodiff	I have not experienced any difficulties due to the COVID-19 pandemic	radio (Matrix)<br>1 Yes<br>0 No
251	covidlf_move	Have you moved from your home because of the COVID-19 pandemic?	yesno<br>1 Yes<br>0 No
252	covidlf_move_why<br>Show the field ONLY if:<br>[covidlf_move] = '1'	If yes, why?	text
253	covidlf_moveloc<br>Show the field ONLY if:<br>[covidlf_move] = '1'	Where did you move to?	radio<br>1 Another house or apartment of my own<br>2 A family member's house or apartment<br>3 A friend's house or apartment<br>4 A health care facility, for example, a nursing home<br>5 A shelter<br>6 Other
254	covidwb_emhlth	Since the beginning of the COVID-19 pandemic in February 2020, my overall emotional health during the pandemic has ...	radio<br>1 Improved<br>2 Stayed the same<br>3 Gotten worse
255	covidwb_sud	Since the beginning of the COVID-19 pandemic in February 2020, has your use of alcohol and/or drugs...	radio<br>1 Increased<br>2 Stayed about the same<br>3 Decreased<br>888 I do not use alcohol or drugs
256	covidwb_lang	Section Header: *Which of the following concerns do you have, as a Native person, about the impacts of the COVID-19 pandemic? Please answer Yes or No for each concern.*<br>It will be harder to practice or learn our Native language	radio (Matrix)<br>1 Yes<br>0 No
257	covidwb_elder	We will lose our elders and wisdom keepers	radio (Matrix)<br>1 Yes<br>0 No
258	covidwb_cult	It will be harder to participate in our cultural practices	radio (Matrix)<br>1 Yes<br>0 No
259	covidwb_tribered	The population of my American Indian or Alaska Native tribe or group will be reduced	radio (Matrix)<br>1 Yes<br>0 No
260	covidwb_stigma	I or my American Indian or Alaska Native tribe or group is being stigmatized, or marked or seen in a negative way	radio (Matrix)<br>1 Yes<br>0 No
261	covidwb_simdis	It reminds me of how our ancestors were wiped out from similar diseases	radio (Matrix)<br>1 Yes<br>0 No
262	covidwb_oth	Other:	radio (Matrix)<br>1 Yes<br>0 No

263	covidwb_noconc	I do not have any concerns, as a Native person, due to the COVID-19 pandemic	radio (Matrix)<br>1 Yes<br>0 No
264	covidwb_oth_desc<br><br>Show the field ONLY if:<br>[covidwb_oth]='1'	Other (please describe):	notes
265	covidwb_disrpt	How disruptive has the COVID-19 pandemic been to your everyday life?	radio<br>1 Not at all disruptive<br>2 A little disruptive<br>3 Somewhat disruptive<br>4 Very disruptive<br>5 Extremely disruptive
266	covidwb_control	Since the beginning of the COVID-19 pandemic, how often have you felt unable to control important things in your life?	radio<br>1 Never<br>2 Rarely<br>3 Sometimes<br>4 Often<br>5 Very often
267	covid19_impact_on_your_well being_complete	Section Header: *Form Status*<br>Complete?	dropdown<br>0 Incomplete<br>1 Unverified<br>2 Complete
Instrument: **COVID-19 Personal Activities** (covid19_personal_activities)			Collapse
268	covidpa_lvwork	In the past month, how many days did you typically leave your home for work or employment?	radio<br>1 Never<br>2 1 to 2 days per week<br>3 3 to 4 days per week<br>4 5 or more days per week<br>888 Not currently working
269	covidpa_lvexer	Section Header: *In the past month, how many days did you typically leave your home for each of the following activities? Please check one answer for each line.*<br>Outdoor exercise and recreation	radio (Matrix)<br>1 Never<br>2 1 to 2 days per week<br>3 3 to 4 days per week<br>4 5 or more days per week
270	covidpa_lverr	Essential errands, for example, going to the grocery store, pharmacy, bank, in-person medical visits, or caretaking for family or friends	radio (Matrix)<br>1 Never<br>2 1 to 2 days per week<br>3 3 to 4 days per week<br>4 5 or more days per week
271	covidpa_lvsoc	Personal and social activities, for example, dining at a restaurant, attending a religious service, visiting family or friends outside your home, or using a gym or fitness center	radio (Matrix)<br>1 Never<br>2 1 to 2 days per week<br>3 3 to 4 days per week<br>4 5 or more days per week
272	covid19_personal_activities_c omplete	Section Header: *Form Status*<br>Complete?	dropdown<br>0 Incomplete<br>1 Unverified<br>2 Complete

Instrument: **COVID-19 Government Response And Trust** (covid19_government_response_and_trust) Collapse

273	covidgvt_resp	Do you think our country's response to the COVID-19 pandemic is...	radio<br>3 Overblown<br>2 Appropriate<br>1 Insufficient
274	covidresp_fed	Section Header: *How would you rate federal, state, local and tribal response to the COVID-19 pandemic? Please check one answer for each line.*<br>Federal health agencies (for example, NIH, CDC, FDA)	radio (Matrix)<br>1 Excellent<br>2 Good<br>3 Fair<br>4 Poor
275	covidresp_ihs	Indian Health Service (IHS)	radio (Matrix)<br>1 Excellent<br>2 Good<br>3 Fair<br>4 Poor
276	covidresp_state	State health department	radio (Matrix)<br>1 Excellent<br>2 Good<br>3 Fair<br>4 Poor
277	covidresp_local	Local or city health department	radio (Matrix)<br>1 Excellent<br>2 Good<br>3 Fair<br>4 Poor
278	covidresp_tribe	Tribal and urban Indian health organizations	radio (Matrix)<br>1 Excellent<br>2 Good<br>3 Fair<br>4 Poor
279	covidcomm_fed	Section Header: *How would you rate federal, state, local and tribal communications about the COVID-19 pandemic? Please check one answer for each line.*<br>Federal health agencies (for example, NIH, CDC, FDA)	radio (Matrix)<br>1 Excellent<br>2 Good<br>3 Fair<br>4 Poor
280	covidcomm_ihs	Indian Health Service (IHS)	radio (Matrix)<br>1 Excellent<br>2 Good<br>3 Fair<br>4 Poor
281	covidcomm_state	State health department	radio (Matrix)<br>1 Excellent<br>2 Good<br>3 Fair<br>4 Poor

282	covidcomm_cty	City health department	radio (Matrix)<br>1 Excellent<br>2 Good<br>3 Fair<br>4 Poor
283	covidcomm_tribe	Tribal and urban Indian health organizations	radio (Matrix)<br>1 Excellent<br>2 Good<br>3 Fair<br>4 Poor
284	covid19_government_response_and_trust_complete	Section Header: *Form Status*<br>Complete?	dropdown<br>0 Incomplete<br>1 Unverified<br>2 Complete
Instrument: **COVID-19 Health Literacy** (covid19_health_literacy)			Collapse
285	covidhl_desc	During the COVID-19 pandemic, it is important to understand how you receive health information.	descriptive
286	trust_pharm	Section Header: *How much do you trust the following sources of COVID-19 related health information? Please check one answer for each line.*<br>Pharmacist	radio (Matrix)<br>1 Strongly disagree<br>2 Disagree<br>3 Agree<br>4 Strongly agree
287	trust_hc	Health care professional, for example, doctor, nurse, or health aide	radio (Matrix)<br>1 Strongly disagree<br>2 Disagree<br>3 Agree<br>4 Strongly agree
288	trust_uihp	Hunter Health Clinic	radio (Matrix)<br>1 Strongly disagree<br>2 Disagree<br>3 Agree<br>4 Strongly agree
289	trust_fam	Family members	radio (Matrix)<br>1 Strongly disagree<br>2 Disagree<br>3 Agree<br>4 Strongly agree
290	trust_frnds	Friends	radio (Matrix)<br>1 Strongly disagree<br>2 Disagree<br>3 Agree<br>4 Strongly agree
291	trust_tv	Television, for example, ABC, CBS, NBC, CNN, FOX, MSNBC	radio (Matrix)<br>1 Strongly disagree<br>2 Disagree<br>3 Agree<br>4 Strongly agree

292	trust_mag	Magazines	radio (Matrix)<br>1 Strongly disagree<br>2 Disagree<br>3 Agree<br>4 Strongly agree
293	trust_news	Newspapers	radio (Matrix)<br>1 Strongly disagree<br>2 Disagree<br>3 Agree<br>4 Strongly agree
294	trust_radio	Radio	radio (Matrix)<br>1 Strongly disagree<br>2 Disagree<br>3 Agree<br>4 Strongly agree
295	trust_socmed	Social media, for example, Twitter, Facebook, blogs	radio (Matrix)<br>1 Strongly disagree<br>2 Disagree<br>3 Agree<br>4 Strongly agree
296	trust_npo	Non-profit organizations	radio (Matrix)<br>1 Strongly disagree<br>2 Disagree<br>3 Agree<br>4 Strongly agree
297	trust_govorg	Government organizations, for example CDC, FDA and NIH	radio (Matrix)<br>1 Strongly disagree<br>2 Disagree<br>3 Agree<br>4 Strongly agree
298	trust_ihs	Indian Health Service or tribal health organization reports or website	radio (Matrix)<br>1 Strongly disagree<br>2 Disagree<br>3 Agree<br>4 Strongly agree
299	trust_relig	Religious or spiritual leaders	radio (Matrix)<br>1 Strongly disagree<br>2 Disagree<br>3 Agree<br>4 Strongly agree
300	trust_tribe	Tribal leaders	radio (Matrix)<br>1 Strongly disagree<br>2 Disagree<br>3 Agree<br>4 Strongly agree

301	wearmask	How often do you wear a mask that covers your nose and mouth when in stores and other indoor public places?	radio<br>6 All of the time<br>5 Most of the time<br>4 More than half the time<br>3 Half the time<br>2 Less than half the time<br>1 Rarely or never
302	pplwearmask	How often do people in your area wear masks that cover their nose and mouth when in stores and other indoor public places?	radio<br>6 All of the time<br>5 Most of the time<br>4 More than half the time<br>3 Half the time<br>2 Less than half the time<br>1 Rarely or never
303	isolate_maintain_job	If you were to test positive for COVID-19, would you be able to isolate without losing your job?	yesno<br>1 Yes<br>0 No
304	quarantine_maintain_job	If you were exposed to someone with COVID-19, would you be able to quarantine without losing your job?	yesno<br>1 Yes<br>0 No
305	covid19_health_literacy_complete	Section Header: *Form Status*<br>Complete?	dropdown<br>0 Incomplete<br>1 Unverified<br>2 Complete
Instrument: **Structural Barriers To COVID-19 Testing** (structural_barriers_to_covid19_testing) Collapse			
306	clinic_trvdiff	How easy or difficult is it for you currently to travel to your clinic for an appointment?	radio<br>1 Very difficult<br>2 Somewhat difficult<br>3 Somewhat easy<br>4 Very easy
307	clinic_hrs	How easy or difficult is it for you to go to the clinic during the hours it is open?	radio<br>1 Very difficult<br>2 Somewhat difficult<br>3 Somewhat easy<br>4 Very easy
308	covid_testcost	How concerned are you about the cost of getting a test for COVID-19?	radio<br>1 Not at all<br>2 A little<br>3 Somewhat<br>4 Very concerned
309	clinic_infect	How concerned are you about getting infected with COVID-19 during an in-person appointment at your clinic?	radio<br>1 Not at all<br>2 A little<br>3 Somewhat<br>4 Very concerned

310	avail_drvthru	Section Header: *How likely would you be to get tested for COVID-19 if the following strategies were available? Please check one answer for each line.*<br>Drive-through testing at or near your clinic	radio (Matrix)<br>1 Extremely Likely<br>2 Very Likely<br>3 Moderately Likely<br>4 Slightly Likely<br>5 Not at all Likely
311	avail_socdist	Increasing the amount of social distancing at the clinic	radio (Matrix)<br>1 Extremely Likely<br>2 Very Likely<br>3 Moderately Likely<br>4 Slightly Likely<br>5 Not at all Likely
312	avail_mobile	A mobile testing unit available closer to where you live	radio (Matrix)<br>1 Extremely Likely<br>2 Very Likely<br>3 Moderately Likely<br>4 Slightly Likely<br>5 Not at all Likely
313	avail_fam	You were tested at the same time as your family or friends	radio (Matrix)<br>1 Extremely Likely<br>2 Very Likely<br>3 Moderately Likely<br>4 Slightly Likely<br>5 Not at all Likely
314	avail_incent	You were paid an incentive for being tested	radio (Matrix)<br>1 Extremely Likely<br>2 Very Likely<br>3 Moderately Likely<br>4 Slightly Likely<br>5 Not at all Likely
315	avail_socserv	Someone at your clinic could help you with social services	radio (Matrix)<br>1 Extremely Likely<br>2 Very Likely<br>3 Moderately Likely<br>4 Slightly Likely<br>5 Not at all Likely
316	avail_famloc	Testing was available at a place in the community that was safe and familiar to you	radio (Matrix)<br>1 Extremely Likely<br>2 Very Likely<br>3 Moderately Likely<br>4 Slightly Likely<br>5 Not at all Likely
317	avail_easy	The test was simple, quick, and easily administered	radio (Matrix)<br>1 Extremely Likely<br>2 Very Likely<br>3 Moderately Likely<br>4 Slightly Likely<br>5 Not at all Likely

318	avail_sameday	Test results were available on the same day	radio (Matrix)<br>1 Extremely Likely<br>2 Very Likely<br>3 Moderately Likely<br>4 Slightly Likely<br>5 Not at all Likely
319	avail_free	The test was free	radio (Matrix)<br>1 Extremely Likely<br>2 Very Likely<br>3 Moderately Likely<br>4 Slightly Likely<br>5 Not at all Likely
320	structural_barriers_to_covid19_testing_complete	Section Header: *Form Status*<br>Complete?	dropdown<br>0 Incomplete<br>1 Unverified<br>2 Complete
Instrument: **Vaccinations** (vaccinations)			Collapse
321	flu_vaccreg	Do you regularly get the annual flu vaccine?	yesno<br>1 Yes<br>0 No
322	covid_vaccine	Have you received a COVID-19 vaccine?	yesno<br>1 Yes<br>0 No
323	safevacc<br>Show the field ONLY if:<br>[covid_vaccine] = '0'	When an effective and safe vaccine for COVID-19 is available to you, how likely will you be to get vaccinated?	radio<br>4 Very likely to get vaccinated<br>3 Somewhat likely to get vaccinated<br>2 Not very likely to get vaccinated<br>1 I would not get vaccinated
324	novacc_risk<br>Show the field ONLY if:<br>[covid_vaccine] = '0'	Section Header: *For each of the following statements about why you might not want to get vaccinated, indicate if it is a major reason, a minor reason, or not a reason at all? Please check one answer for each line.*<br>I am not at risk of getting a serious case of COVID-19	radio (Matrix)<br>3 Major reason I would not get vaccinated<br>2 Minor reason I would not get vaccinated<br>1 Not a reason I would not get vaccinated
325	novacc_notser<br>Show the field ONLY if:<br>[covid_vaccine] = '0'	I do not think the pandemic is as serious as public officials think	radio (Matrix)<br>3 Major reason I would not get vaccinated<br>2 Minor reason I would not get vaccinated<br>1 Not a reason I would not get vaccinated
326	novacc_toolate<br>Show the field ONLY if:<br>[covid_vaccine] = '0'	It will be too late in the pandemic to make getting vaccinated for COVID-19 worthwhile	radio (Matrix)<br>3 Major reason I would not get vaccinated<br>2 Minor reason I would not get vaccinated<br>1 Not a reason I would not get vaccinated
327	novacc_safety<br>Show the field ONLY if:<br>[covid_vaccine] = '0'	I am concerned about safety risks from the COVID-19 vaccine	radio (Matrix)<br>3 Major reason I would not get vaccinated<br>2 Minor reason I would not get vaccinated<br>1 Not a reason I would not get vaccinated

328	novacc_meds<br>Show the field ONLY if:<br>[covid_vaccine] = '0'	If I get COVID-19, I can get medication to treat it	radio (Matrix)<br>3 Major reason I would not get vaccinated<br>2 Minor reason I would not get vaccinated<br>1 Not a reason I would not get vaccinated
329	novacc_hadcov<br>Show the field ONLY if:<br>[covid_vaccine] = '0'	I already had COVID-19	radio (Matrix)<br>3 Major reason I would not get vaccinated<br>2 Minor reason I would not get vaccinated<br>1 Not a reason I would not get vaccinated
330	novacc_exp<br>Show the field ONLY if:<br>[covid_vaccine] = '0'	It will be too expensive for me to get the COVID-19 vaccine	radio (Matrix)<br>3 Major reason I would not get vaccinated<br>2 Minor reason I would not get vaccinated<br>1 Not a reason I would not get vaccinated
331	novacc_loc<br>Show the field ONLY if:<br>[covid_vaccine] = '0'	It is too difficult to get to a place where I can get vaccinated for COVID-19	radio (Matrix)<br>3 Major reason I would not get vaccinated<br>2 Minor reason I would not get vaccinated<br>1 Not a reason I would not get vaccinated
332	novacc_distrust<br>Show the field ONLY if:<br>[covid_vaccine] = '0'	I don't trust public health officials to provide reliable information about the safety of the COVID-19 vaccine	radio (Matrix)<br>3 Major reason I would not get vaccinated<br>2 Minor reason I would not get vaccinated<br>1 Not a reason I would not get vaccinated
333	novacc_drugco<br>Show the field ONLY if:<br>[covid_vaccine] = '0'	I don't trust pharmaceutical or drug companies that make the COVID-19 vaccine to provide reliable information about its safety	radio (Matrix)<br>3 Major reason I would not get vaccinated<br>2 Minor reason I would not get vaccinated<br>1 Not a reason I would not get vaccinated
334	novacc_prsnl<br>Show the field ONLY if:<br>[covid_vaccine] = '0'	I am worried about having to reveal personal information to the government in order to get vaccinated for COVID-19	radio (Matrix)<br>3 Major reason I would not get vaccinated<br>2 Minor reason I would not get vaccinated<br>1 Not a reason I would not get vaccinated
335	novacc_guinea<br>Show the field ONLY if:<br>[covid_vaccine] = '0'	I am worried that American Indian and Alaska Natives will be used as guinea pigs for the COVID-19 vaccine	radio (Matrix)<br>3 Major reason I would not get vaccinated<br>2 Minor reason I would not get vaccinated<br>1 Not a reason I would not get vaccinated
336	novacc_notforaian<br>Show the field ONLY if:<br>[covid_vaccine] = '0'	The COVID-19 vaccine is not specifically made for American Indian and Alaska Native people	radio (Matrix)<br>3 Major reason I would not get vaccinated<br>2 Minor reason I would not get vaccinated<br>1 Not a reason I would not get vaccinated
337	novacc_allergic<br>Show the field ONLY if:<br>[covid_vaccine] = '0'	I'm allergic to vaccines	radio (Matrix)<br>3 Major reason I would not get vaccinated<br>2 Minor reason I would not get vaccinated<br>1 Not a reason I would not get vaccinated
338	novacc_needles<br>Show the field ONLY if:<br>[covid_vaccine] = '0'	I don't like needles	radio (Matrix)<br>3 Major reason I would not get vaccinated<br>2 Minor reason I would not get vaccinated<br>1 Not a reason I would not get vaccinated
339	yesvacc_famsafe<br>Show the field ONLY if:<br>[covid_vaccine] = '0'	Section Header: *For each of the following statements about why you would get vaccinated, indicate if it is a major reason, a minor reason, or not a reason at all? Please check one answer for each line.*<br>I want to keep my family safe	radio (Matrix)<br>3 Major reason I would get vaccinated<br>2 Minor reason I would get vaccinated<br>1 Not a reason I would get vaccinated

340	yesvacc_commsafe<br>Show the field ONLY if:<br>[covid_vaccine] = '0'	I want to keep my community safe	radio (Matrix)<br>3 Major reason I would get vaccinated<br>2 Minor reason I would get vaccinated<br>1 Not a reason I would get vaccinated
341	yesvacc_selfsafe<br>Show the field ONLY if:<br>[covid_vaccine] = '0'	I want to keep myself safe	radio (Matrix)<br>3 Major reason I would get vaccinated<br>2 Minor reason I would get vaccinated<br>1 Not a reason I would get vaccinated
342	yesvacc_hlthprb<br>Show the field ONLY if:<br>[covid_vaccine] = '0'	I have a chronic health problem, like asthma or diabetes	radio (Matrix)<br>3 Major reason I would get vaccinated<br>2 Minor reason I would get vaccinated<br>1 Not a reason I would get vaccinated
343	yesvacc_dr<br>Show the field ONLY if:<br>[covid_vaccine] = '0'	My doctor told me to get a COVID-19 vaccine	radio (Matrix)<br>3 Major reason I would get vaccinated<br>2 Minor reason I would get vaccinated<br>1 Not a reason I would get vaccinated
344	yesvacc_sick<br>Show the field ONLY if:<br>[covid_vaccine] = '0'	I don't want to get really sick from COVID-19	radio (Matrix)<br>3 Major reason I would get vaccinated<br>2 Minor reason I would get vaccinated<br>1 Not a reason I would get vaccinated
345	yesvacc_safeothers<br>Show the field ONLY if:<br>[covid_vaccine] = '0'	I want to feel safe around other people	radio (Matrix)<br>3 Major reason I would get vaccinated<br>2 Minor reason I would get vaccinated<br>1 Not a reason I would get vaccinated
346	yesvacc_normal<br>Show the field ONLY if:<br>[covid_vaccine] = '0'	I believe life won't go back to normal until most people get a COVID-19 vaccine	radio (Matrix)<br>3 Major reason I would get vaccinated<br>2 Minor reason I would get vaccinated<br>1 Not a reason I would get vaccinated
347	vacc_info<br>Show the field ONLY if:<br>[covid_vaccine] = '0'	What information would you want to decide whether to get a COVID-19 vaccine?	notes
348	vacc_study	How willing would you be to participate in a COVID-19 vaccine study?	radio<br>1 Not willing<br>2 Somewhat unwilling<br>3 Somewhat willing<br>4 Very willing
349	vaccstud_univ	Section Header: *Please rate your willingness to enroll in a COVID-19 vaccine study based on the extra details below. Please consider each question separately from the others. Please check one answer for each line.*<br>If the study was led by someone from a university?	radio (Matrix)<br>1 Not willing<br>2 Somewhat unwilling<br>3 Somewhat willing<br>4 Very willing
350	vaccstud_fedgov	If the study was led by the federal government?	radio (Matrix)<br>1 Not willing<br>2 Somewhat unwilling<br>3 Somewhat willing<br>4 Very willing

351	vaccstud_natsci	If the study was led by a Native scientist?	radio (Matrix)<br>1 Not willing<br>2 Somewhat unwilling<br>3 Somewhat willing<br>4 Very willing
352	vaccstud_commdes	If members of your community helped to design the study?	radio (Matrix)<br>1 Not willing<br>2 Somewhat unwilling<br>3 Somewhat willing<br>4 Very willing
353	vaccstud_sick	If you had a loved one who was or is sick with COVID-19?	radio (Matrix)<br>1 Not willing<br>2 Somewhat unwilling<br>3 Somewhat willing<br>4 Very willing
354	vaccstud_riskinfo	If you received information about your risk of getting sick with COVID-19 as part of the study?	radio (Matrix)<br>1 Not willing<br>2 Somewhat unwilling<br>3 Somewhat willing<br>4 Very willing
355	vaccstud_commben	If you knew the results would benefit your community?	radio (Matrix)<br>1 Not willing<br>2 Somewhat unwilling<br>3 Somewhat willing<br>4 Very willing
356	vaccstud_consent	If you trusted that your information would never be used for anything without your consent?	radio (Matrix)<br>1 Not willing<br>2 Somewhat unwilling<br>3 Somewhat willing<br>4 Very willing
357	vaccstud_tribalenc	If your tribal and community leaders encouraged people to participate?	radio (Matrix)<br>1 Not willing<br>2 Somewhat unwilling<br>3 Somewhat willing<br>4 Very willing
358	vaccstud_freehc	If you got free health care if you became sick with COVID-19 or had side effects from the vaccine?	radio (Matrix)<br>1 Not willing<br>2 Somewhat unwilling<br>3 Somewhat willing<br>4 Very willing
359	vaccinations_complete	Section Header: *Form Status*<br>Complete?	dropdown<br>0 Incomplete<br>1 Unverified<br>2 Complete
